# Amoeba Predation of Cryptococcus neoformans Results in Pleiotropic Changes to Traits Associated with Virulence

**DOI:** 10.1128/mBio.00567-21

**Published:** 2021-04-27

**Authors:** Man Shun Fu, Livia C. Liporagi-Lopes, Samuel R. dos Santos, Jennifer L. Tenor, John R. Perfect, Christina A. Cuomo, Arturo Casadevall

**Affiliations:** a Department of Molecular Microbiology and Immunology, Johns Hopkins Bloomberg School of Public Health, Baltimore, Maryland, USA; b Departamento de Análises Clínicas e Toxicológicas, Faculdade de Farmácia, Universidade Federal do Rio de Janeiro, Rio de Janeiro, Brazil; c Departamento de Microbiologia, Instituto de Ciências Biomédicas, Universidade de São Paulo, São Paulo, Brazil; d Division of Infectious Diseases, Department of Medicine and Department of Molecular Genetics and Microbiology, Duke University, Durham, North Carolina, USA; e Infectious Disease and Microbiome Program, Broad Institute, Cambridge, Massachusetts, USA; University of Michigan and VA Ann Arbor Health Systems; Texas Christian University

**Keywords:** *Acanthamoeba castellanii*, amoeba, *Cryptococcus neoformans*, evolution, host-pathogen interactions, macrophages, opportunistic fungi

## Abstract

Amoeboid predators, such as amoebae, are proposed to select for survival traits in soil microbes such as Cryptococcus neoformans; these traits can also function in animal virulence by defeating phagocytic immune cells, such as macrophages. Consistent with this notion, incubation of various fungal species with amoebae enhanced their virulence, but the mechanisms involved are unknown. In this study, we exposed three strains of C. neoformans (1 clinical and 2 environmental) to predation by Acanthamoeba castellanii for prolonged times and then analyzed surviving colonies phenotypically and genetically. Surviving colonies comprised cells that expressed either pseudohyphal or yeast phenotypes, which demonstrated variable expression of traits associated with virulence, such as capsule size, urease production, and melanization. Phenotypic changes were associated with aneuploidy and DNA sequence mutations in some amoeba-passaged isolates, but not in others. Mutations in the gene encoding the oligopeptide transporter (CNAG_03013; *OPT1*) were observed among amoeba-passaged isolates from each of the three strains. Isolates derived from environmental strains gained the capacity for enhanced macrophage toxicity after amoeba selection and carried mutations on the CNAG_00570 gene encoding Pkr1 (AMP-dependent protein kinase regulator) but manifested reduced virulence in mice because they elicited more effective fungal-clearing immune responses. Our results indicate that C. neoformans survival under constant amoeba predation involves the generation of strains expressing pleiotropic phenotypic and genetic changes. Given the myriad potential predators in soils, the diversity observed among amoeba-selected strains suggests a bet-hedging strategy whereby variant diversity increases the likelihood that some will survive predation.

## Abstract (precis)

Cryptococcus neoformans is a ubiquitous environmental fungus that is also a leading cause of fatal fungal infection in humans, especially among immunocompromised patients. A major question in the field is how an environmental yeast such as C. neoformans becomes a human pathogen when it has no need for an animal host in its life cycle.

## INTRODUCTION

Cryptococcus neoformans is a major, life-threatening fungal pathogen that predominantly infects severely immunocompromised patients and causes over 180,000 deaths per year worldwide (1). C. neoformans expresses virulence factors that promote its pathogenicity in humans, including formation and enlargement of a polysaccharide capsule that interferes with the host immune system in varied ways, melanin production that protects against oxidative stress (2–5), and extracellular secretion of various enzymes, including phospholipase and urease (6, 7). C. neoformans is found primarily and ubiquitously in environments such as soils contaminated with bird excreta or from trees (8–11). It is a saprophyte and does not require an animal host for survival and reproduction. Besides, there is rare evidence of human-to-human transmission and thus it is unlikely that its virulence traits were selected for causing disease in humans or animals. That raises the fundamental question of how C. neoformans acquired those traits, which are essential for pathogenesis of cryptococcosis in human.

To explain this enigma, the “amoeboid predator-fungal animal virulence” hypothesis posits that virulence emerges accidentally from coincident selection resulting from selective pressures in both natural environmental and animal niches such as predatory amoebae and nematodes (12). According to this view, microbial traits selected for environmental survival also confer the capacity for virulence by promoting survival in animal hosts (12, 13). For example, the capsule can protect the fungi from desiccation and against predatory amoebae (14, 15), while melanin may reduce damage of fungi from exposure to UV radiation, osmotic stresses, or extreme temperatures (16–19). Urease plays a nutritional role in nitrogen acquisition in the environment (20). Moreover, it is striking that C. neoformans isolates from the soil are virulent for animal hosts (21). Understanding the evolutionary adaption of C. neoformans in nature will help us to understand further the origin of virulence and pathogenesis of cryptococcosis.

Amoebae are one of the major sources of selective pressure in nature for a broad range of soil microorganisms that have pathogenic potential for humans, including bacteria such as Legionella pneumophila and Mycobacterium spp. and fungi such as Cryptococcus neoformans, Aspergillus fumigatus, and Paracoccidioides spp. (13, 15, 22–24). Similarly to human macrophages, amoebae ingest microorganisms; undergo a respiratory burst, phagosome maturation, and acidification; and express cell surface receptors and expel undigested materials (25–31). However, many bacteria and fungi have strategies to survive in amoebae that function in parallel for survival in mammalian phagocytic cells. For example, L. pneumophila utilizes similar cellular and molecular mechanisms of invasion, survival, and replication inside both amoebae and macrophages (32–37). Amoeba-grown L. pneumophila bacteria are more invasive for epithelial cells and macrophages (22). After passage in amoebae, Mycobacterium avium enhances both entry and intracellular replication in epithelial cells and is more virulent in the macrophage and mouse models of infection (23). Among fungal pathogens, concordance of virulence factor function for amoebae and animals was also demonstrated for A. fumigatus (13). For example, the mycotoxin fumagillin can inhibit the growth of Entamoeba histolytica, and it can also cause mammalian epithelial cell damage (38). Many studies have been done to explore amoeba-C. neoformans interaction and have shown evidence that amoebae influence the virulence of C. neoformans for mammalian infection (39, 40).

Acanthamoeba castellanii was originally isolated from cultures of a Cryptococcus sp. and, like other amoeba species, it preys on *Cryptococcus* spp. (41, 42). There is evidence that amoebae are natural predators of C. neoformans in the natural environment (43). On the other hand, C. neoformans is able to resist destruction by amoebae, especially in nutrient-poor conditions (44) without metal cations (45). Several studies have shown that the virulence factors and the cellular process that fungi use for defending against amoeba predation are remarkably similar to those employed for mammalian virulence. For example, capsule formation and melanin production are important for C. neoformans to resist predation by A. castellanii and play important roles for pathogenicity in mammalian infection (15, 39). Interestingly, the phospholipids that are secreted by both macrophages and amoebae trigger capsule enlargement (40). The nonlytic exocytosis process which is found in macrophage-containing C. neoformans can be also observed in A. castellanii and Dictyostelium discoideum through the similar action of actin polymerization (46, 47). Transcriptional studies showed a conserved metabolic response of C. neoformans to the microenvironments of both macrophages and amoebae (48). All of those common strategies found to adapt to both amoebae and macrophages support the hypothesis that cryptococcal pathogenesis is derived from the interaction with amoebae in the natural environment. More direct evidence comes from an experiment on the passage of an attenuated cryptococcal strain to D. discoideum cultures that showed enhancement of fungal virulence in a murine infection model (39). Passaged C. neoformans also exhibits capsule enlargement and rapid melanization, suggesting that those are mechanisms to enhance the survival of fungus in mice. However, the underlying mechanism of how these phenotypic changes occur is still unclear.

In this study, we sought to determine the long-term evolutionary adaption of C. neoformans when interacting with amoebae and whether the adaption affected virulence traits for animal hosts. Our results show that persistent amoeba predation was associated with the emergence of pleiotropic phenotypic changes of C. neoformans.

## RESULTS

### Selection of amoeba resistance strains.

**FIG 1:**
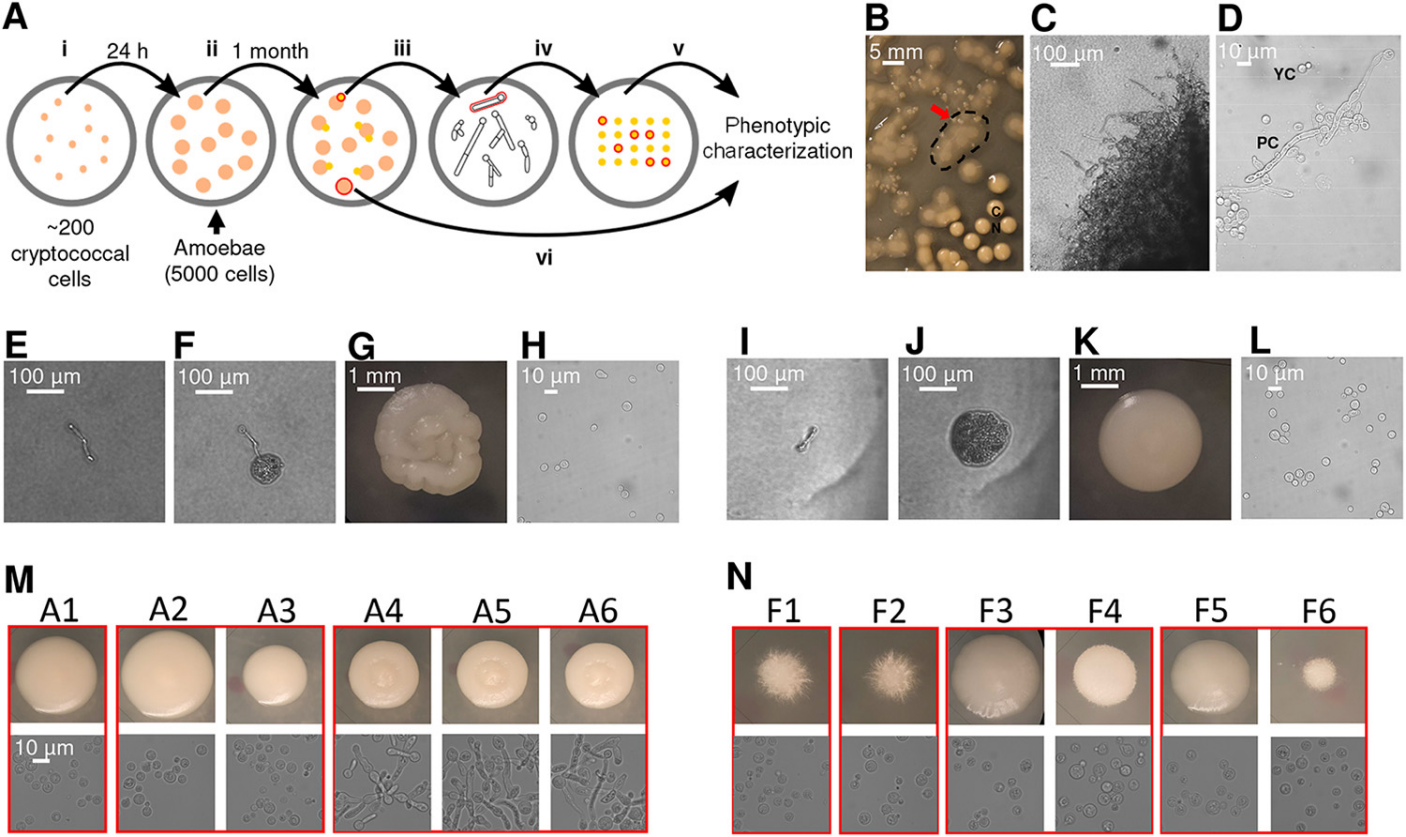
C. neoformans colonies exhibit various cellular and colony morphologies after coincubation with amoebae in Sabouraud agar. (A) Schematic representation of experimental setup for amoeba resistance strain selection. (i) Approximately 200 cryptococcal cells were spread on agar. (ii) After 24 h, approximately 5,000 A. castellanii cells were placed on agar with cryptococcal colonies. (iii) After approximately 1 month of coincubation, small colonies emerged within the predation zone of A. castellanii. Surviving colonies were picked using pipette tips and transferred to phosphate-buffered saline (PBS) in a 3-mm culture dish. (iv) A total of 20 individual hyphae from 2 to 4 colonies were selected under a microscope and transferred to fresh Sabouraud agar. Plates were incubated at 30°C to generate colonies. (v) Six colonies were then selected for further phenotypic characterization. (vi) Control colonies were also picked from the same plate of hyphal isolates but without interaction with amoeba. (B) Small colonies of H99 cells (red arrow) surviving in a mat of amoebae that appears as a hazy, cloudy area (denoted by dashed line). Typical C. neoformans colonies (CN) are visible on the bottom right of the image. (C) Cells in the surviving colony exhibit hyphal or pseudohyphal morphology (×100 magnification). (D) Both pseudohyphae and yeast cells were identified on a wet mount of samples taken from the surviving colony (×400). (E) Single pseudohyphal cell isolated from the surviving colonies and transferred onto a fresh amoeba-free solid medium, where it formed new colonies. (F) Microcolony with primarily yeast cells, formed from a single pseudohyphal cell in 24 h. (G) The colony developed a serrated appearance after 2 days. (H) Yeast cells were identified on a wet mount of samples taken from the serrated colony. (I to L) Images show another example of single pseudohyphal cell isolation. A smooth colony was formed from this particular pseudohyphal cell. (M and N) The same experiment was performed on environmental strains A1-35-8 and Ftc-555-1. Various cellular and colony morphologies have been identified among isolates A1 to A6 and F1 to F6 in backgrounds of A1-35-8 and Ftc555-1, respectively. Colonies grown from individual hyphae that were isolated from the same surviving colony are grouped in red boxes.

We studied the interaction between C. neoformans and Acanthamoeba castellanii by culturing them together on Sabouraud agar. For the initial experiments, we used the well-studied common laboratory strain H99. The experimental setup involved spreading approximately 200 cryptococcal cells on agar, followed by placing approximately 5,000 A. castellanii cells on the plate (Fig. 1A). After approximately 1 month of coincubation, small colonies emerged within the predation zone of A. castellanii (Fig. 1A and B), sometimes under the mat of amoebae. Microscopic morphological analysis of cells in those colonies revealed pseudohyphal and hyphal forms of C. neoformans (Fig. 1C and D). We selected 20 single hyphal cells from two colonies (10 hyphae from each colony), and these were transferred to a fresh Sabouraud agar plate without amoeba (Fig. 1A, E, and I). After 24 h, microcolonies composed exclusively of yeast cells emerged on the agar (Fig. 1F and J), which manifested two distinct colony morphologies, smooth and serrated, after 2 days of agar growth (Fig. 1G and K). All of the cells from these colonies were yeasts (Fig. 1H and L). The same experiment was then repeated with two environmental C. neoformans strains, A1-35-8 and Ftc555-1. A1-35-8 with a genotype of the VN1 molecular type was isolated from pigeon guano in the United States, while Ftc555-1 was isolated from a mopane tree in Botswana and had the VNB molecular type. Both strains were avirulent in mouse model (49–51). A total of 20 single hyphae were picked from four surviving colonies (five hyphae from each colony) to a fresh agar plate. Like the experience with H99, these strains responded to the presence of amoebae by generating cells that formed colonies with various cellular and colony morphologies, of which some (isolates A4 to A6) were slightly serrated, with pseudohyphal cells (Fig. 1M). We also observed some hyphal colonies formed by Ftc555-1 cells, but these eventually converted back to yeast cells when streaked on fresh agar medium (Fig. 1N). The results showed that after interacting with amoebae, C. neoformans can develop a large variety of cellular and colony morphologies even in amoeba-free medium.

**FIG 2:**
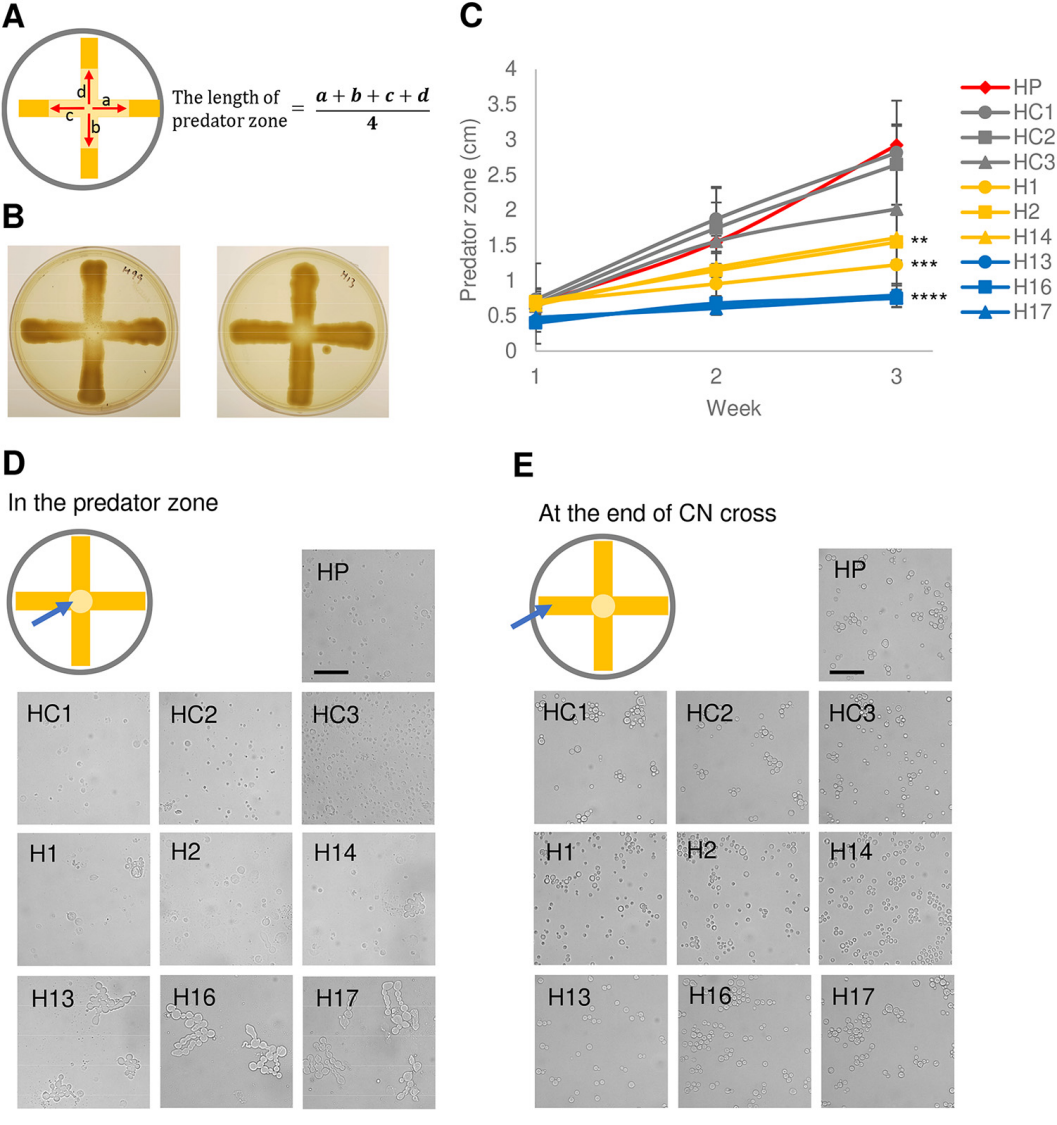
Isolates in H99 background derived from exposure to amoebae demonstrated increased resistance to amoeba killing by rapid pseudohyphal formation. (A) Scheme of amoeba killing assay. C. neoformans was streaked in a cross, while A. castellanii was dropped at the intersection of the cross on Sabouraud agar. Data shown are the average of the distance between boundary and center of the clear predation zone in four indicated directions (a and b), with the area being the predation zone. (B) Representative images of amoeba killing assay on agar plates. Images were taken 3 weeks after A. castellanii was dropped at the intersection of C. neoformans culture. Left, H99; right, H13. (C) All of the isolates that had prior exposure to amoebae had smaller clear zones than those of their parental strain and controls, consistent with enhanced resistance. HP, parental strain; HC1-3, controls; H, isolates derived from H99 after exposure to amoeba. Data are means from three biological replicates, and error bars are standard deviation (SD). **, *P < *0.01; ***, *P < *0.001; ****, *P < *0.0001, compared to parental strain by one-way analysis of variance (ANOVA) and followed by Tukey’s multiple-comparison test. (D) Samples were taken from the peripheral areas of the predation zone after 1 week of coincubation with amoebae and visualized under a microscope. All of the isolates showed pseudohyphal formation, but the parental strain and the controls did not. (E) Sample were taken from the end of the cross, where C. neoformans have not yet contacted A. castellanii. All of the isolates manifested yeast cell morphology.

Six colonies from each strain were selected together with three controls, which were colonies on the same plate with isolates but without interacting with amoebae, for further phenotypic characterization (Fig. 1A). These will be referred to here as amoeba-passaged isolates with numbers preceded by the letters H, A, and F to indicate their origins from strains H99, A1-35-8, and Ftc555-1, respectively. Controls will be referred to as HC, AC, and FC, and parental strains will be referred to as HP, AP, and FP. Amoeba-passaged isolates, their parental strains, and controls are listed in Table S1 in the supplemental material. To test if amoebae exert selection pressure that resulted in amoeba-resistant cells, we examined if those isolates increased their survival during amoeba interaction. Isolates were then coincubated with amoebae in the agar medium again, with C. neoformans streaked into a cross pattern, and amoebae were spotted in the center, at the intersection of the two perpendicular streaks (Fig. 2A and B). The radii of clear zones were measured as a function of time, and these represented how well the amoebae cleared the culture of C. neoformans. All of the amoeba-passaged isolates derived from H99 had a reduced size of predation zone compared to those of their controls and parental strain (Fig. 2C). In particular, the isolates that formed smooth colonies (H13, H16, and H17) had the smallest predation zone (Fig. 2C). This result implies that amoeba passage resulted in C. neoformans strains with an increased ability to subsequently resist predation by amoebae. Next, we investigated the mechanism of the resistance. Samples were taken at the edge of the predation zone at the early stage of the interaction (week 1), and observed under microscope. Isolates H13, H16, and H17 formed pseudohyphae, while most of the cells in isolates H1, H2, and H14 were in yeast form, with some displaying pseudohyphae (Fig. 2D). However, no pseudohyphae were found in cells from controls and H99 parental colonies, although pseudohyphae were eventually formed at the late stage of the interaction. Samples were also taken at a distance from the predation zone, where cryptococcal cells had no contact with amoebae, and in each of these regions all cells were in yeast form (Fig. 2E). These results showed that pseudohyphal cells emerged rapidly from each of the amoeba-passaged strains, even though their cells were yeast prior to the incubation with amoebae, and that pseudohyphal formation is a major mechanism of increased ability to resist predation.

10.1128/mBio.00567-21.1 TABLE S1 List of amoeba-passaged isolates and their parental strains analyzed in this study. Download Table S1, DOCX file, 0.01 MB. Copyright © 2021 Fu et al. 2021 Fu et al. https://creativecommons.org/licenses/by/4.0/ This content is distributed under the terms of the Creative Commons Attribution 4.0 International license.

**FIG 3:**
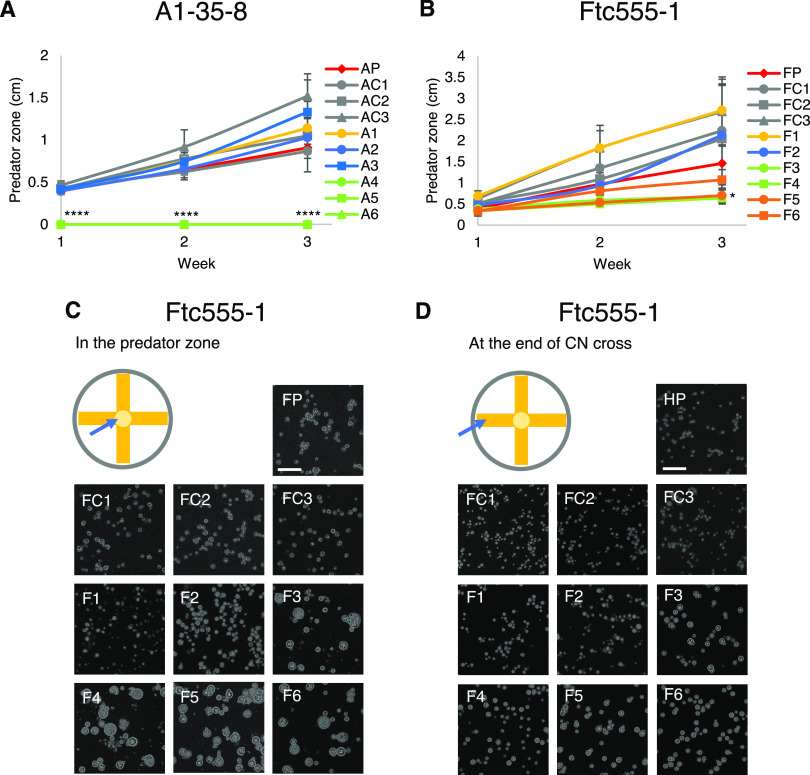
Some of the isolates recovered from the environmental strains A1-35-8 and Ftc-555-1 exhibited increased resistance to A. castellanii. (A) No clear predation zone of clearance was apparent with isolates A4 to A6, while larger predation zones were apparent for isolates A1 to A3 compared to those of their parental strains. AP, parental strain; AC1 to AC3, controls; A1 to A6, isolates derived from A1-35-8 after exposure to amoebae. ****, *P < *0.0001, compared to parental strain by one-way ANOVA and followed by Tukey’s multiple-comparison test. (B) Isolates F3 to F5 showed a smaller predation zone than that of their parental strain. Data are means from three biological replicates, and error bars are SD. FP, parental strain; FC1 to FC3, controls; F1 to F6, isolates derived from Ftc555-1 after exposure to amoebae. *, *P < *0.1, compared to parental strain by one-way ANOVA and followed by Tukey’s multiple-comparison test. (C) Ftc-555-1 samples were collected from the predation zone after 1 week of coincubation. Isolates F3 to F6 formed larger-sized cells than those of their parental strain and controls. (D) The cell size of isolates F3 to F6 from the end of the cross is slightly larger than those of parental strain and controls, but they are not as large as the cells taken from the predation zone.

When the isolates derived from A1-35-8 and Ftc555-1 strains were again exposed to A. castellanii, some but not all exhibited increased resistance to amoebae (Fig. 3A and B). Isolates derived from A1-35-8 (A4 to A6) were significantly more resistant than the others (Fig. 3A). That may be due to maintenance of pseudohyphal cell morphology by isolates A4 to A6 even in the amoeba-free medium. Isolates F3 to F5 manifested increased resistance to amoeba but, unlike the H99-derived isolates, displayed no pseudohyphal formation but had larger cells compared to their parental strain (Fig. 3C and D) at the early stage of interaction, which may be another survival strategy for C. neoformans against amoebae. In this regard, phagocytosis of C. neoformans by macrophage was reduced by cell enlargement of C. neoformans (52–54). The resistance of isolates F3 to F5 to amoebae may reflect their larger cell size.

### Effects of amoeba selection on known virulence factors.

**FIG 4:**
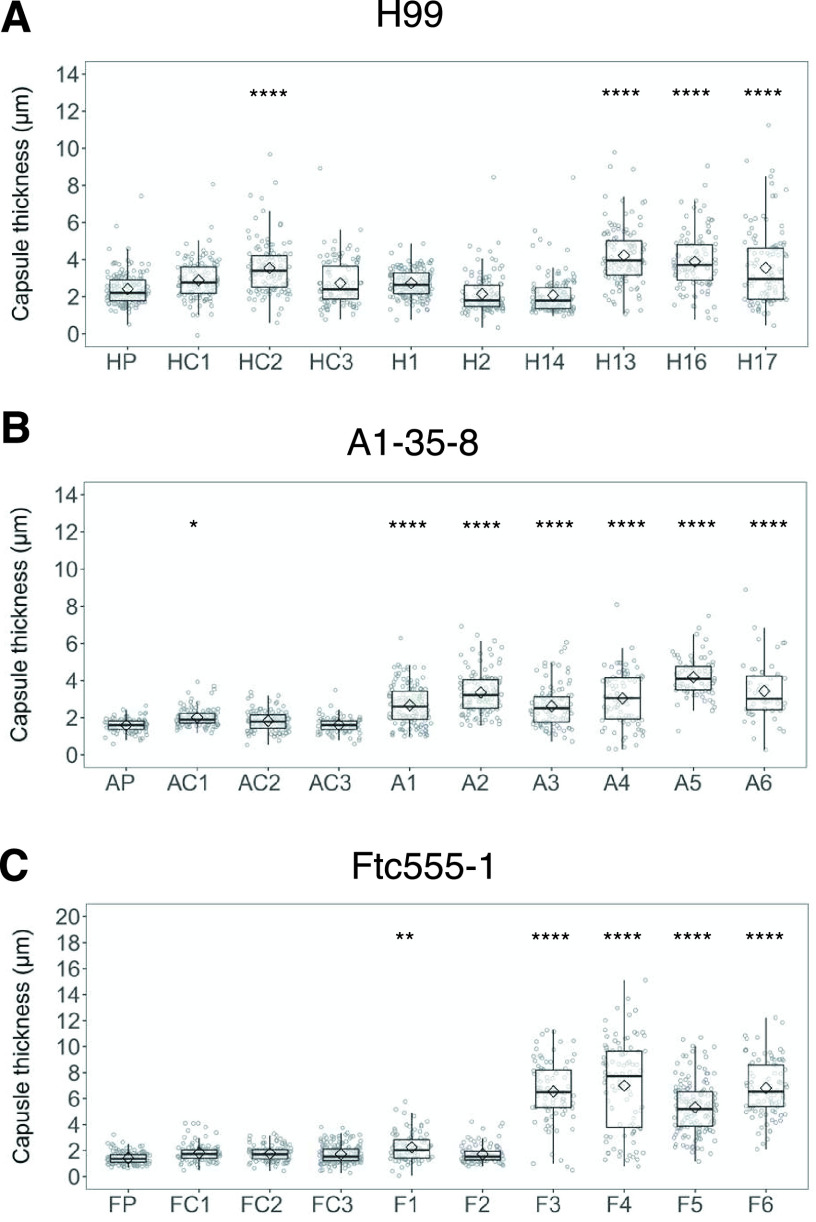
Capsule thickness for cells of the parent strain and amoeba-selected strains. (A) H99 isolates (B), A1-35-8 isolates, and (C) Ftc555-1 isolates were cultured in minimal medium at 30°C for 3 days. Capsule was visualized by counterstaining with India ink. HP, AP, FP: parental strains; HC, AC, FC: controls. Boxes indicate the 25th and 75th percentiles; bars show the 5th and 95th percentiles. The line and diamond within each box indicate the median and mean, respectively. *, *P < *0.1; **, *P < *0.01; ****, *P < *0.0001; one-way ANOVA, followed by Tukey’s multiple-comparison test.

**FIG 5:**
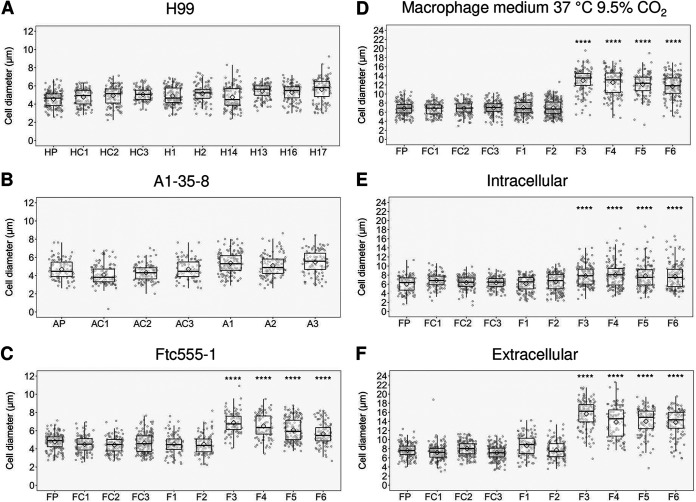
Cellular dimensions for cells of parent strain and amoeba-selected strains. (A) H99 isolates (B), A1-35-8 isolates, and (C) Ftc555-1 isolates were cultured in minimal medium for 3 days. HP, AP, FP: parental strains; HC, AC, FC: controls. (D to E) Ftc555-1 isolates were also cultured in macrophage medium and with bone marrow-derived macrophages (BMDMs) at 37°C and 9.5% CO_2_ for 24 h. Extracellular cryptococcal cells were collected from the culture supernatant, while intracellular cells were retrieved from lysing the BMDMs. HP, AP, FP: parental strains; HC, AC, FC: controls. Boxes indicate the 25th and 75th percentiles; bars show the 5th and 95th percentiles. The line and diamond within each box indicate the median and mean, respectively. ****, *P < *0.0001; one-way ANOVA, followed by Tukey’s multiple-comparison test.

**FIG 6:**
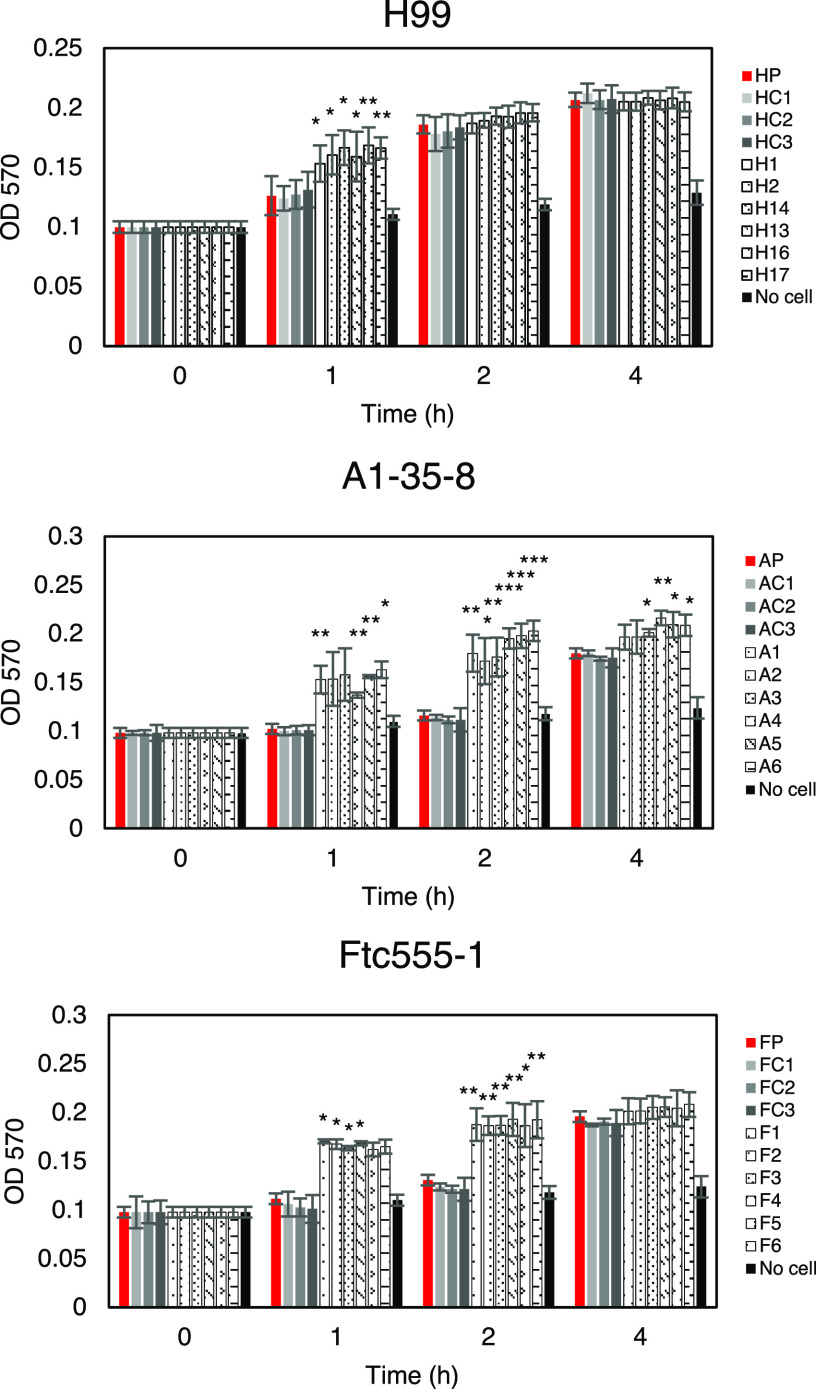
Urease activity for cells of the parent strain and amoeba-selected strains. The urease activity of cryptococcal cells were detected by using the rapid urea broth (RUH) method. Amoeba-passaged isolates are labeled with numbers preceded by the letters H, A, and F to indicate their origin from strains H99, A1-35-8, and Ftc555-1, respectively. HP, AP, FP: parental strains; HC, AC, FC: controls. The assay was performed in triplicate for each time point. Error bars represent SD. *, *P < *0.1; **, *P < *0.01; ***, *P < *0.001; unpaired *t* test. OD_570_, optical density at 570 nm.

**FIG 7:**
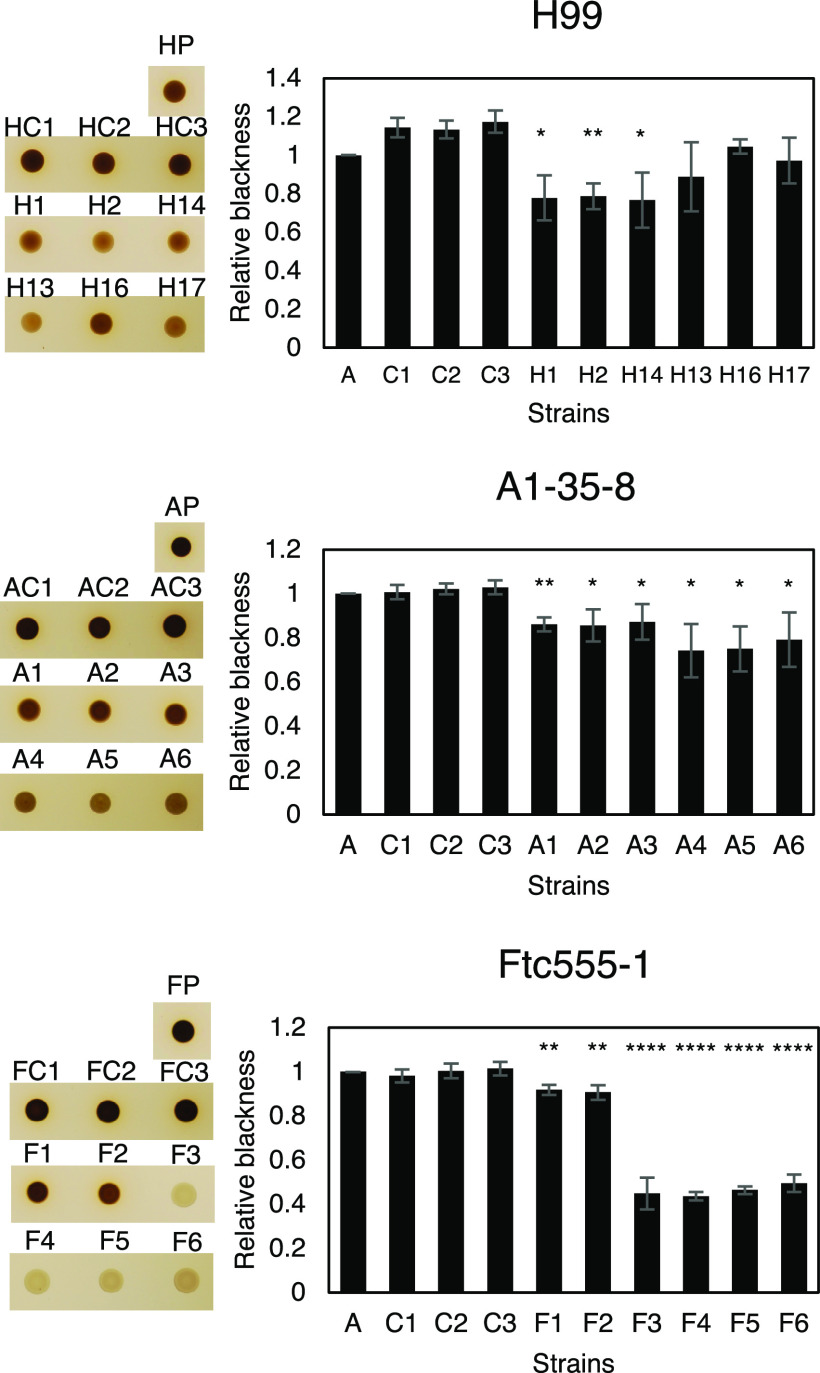
Melanization for cells of parent strain and amoeba-selected strains. Melanization was analyzed by spotting the 10^6^ cryptococcal cells on minimal medium agar with l-3,4-dihydroxyphenylalanine (l-DOPA) for 24 h. The pigmentation of colonies was measured through grayscale pixel quantification using the software ImageJ. Relative blackness was calculated as a ratio of grayscale quantification between isolates, their parental strains (HP, AP, and FP), and controls (HC, AC, and FC). Error bars represent SD. *, *P < *0.1; **, *P < *0.01; ****, *P < *0.0001; unpaired *t* test.

C. neoformans expresses virulence factors that promote its pathogenicity, including formation and enlargement of a polysaccharide capsule, melanin production, extracellular secretion of urease, and cell enlargement. To evaluate whether the emergence of variant forms of C. neoformans was accompanied by changes to known virulence factors, we analyzed the virulence-related phenotypic characteristics of the isolates derived from the three strains. Isolates H13, H16, and H17 and each of the isolates derived from the A1-35-8 strain had larger capsule thickness relative to those of their parental strain when cultured in minimal medium but cell sizes were similar, while isolates F3 to F6 increased both their capsule and cell sizes (Fig. 4 and 5). Isolates F3 to F6 also had more cells with a size larger than 10 μm inside macrophages (Fig. 5E; see also Fig. S1 in the supplemental material). Time-lapse imaging was also done to confirm that evolved strain of Ftc-555-1 was able to increase its cell size after engulfment by macrophages (see Movies S1 and S2 in the supplemental material). Cell enlargement of isolates F3 to F6 has been also observed extracellularly in macrophage medium at 37°C and 9.5% CO_2_ (Fig. 5D and F and Fig. S1), showing that this response is not specific to ingestion by macrophages. All of the isolates had increased urease activity compared to that of their parental strain (Fig. 6). Isolates H1, H2, and H14 and all of the isolates of A1-35-8 and Ftc555-1 manifested less melanin production (Fig. 7).

10.1128/mBio.00567-21.3 FIG S1 Representative images of cellular dimensions for cells of Ftc555-1 and its amoeba-selected strains (F3 to F6). Ftc555-1 isolates have also been cultured in macrophage medium and with bone marrow-derived macrophages (BMDMs) at 37°C and 9.5% CO_2_ for 24 h. Extracellular cryptococcal cells were collected from the culture supernatant, while intracellular cells were retrieved from lysing the BMDM. The cells were counterstained with India ink. FP, Ftc555-1. Download FIG S1, PDF file, 2.0 MB. Copyright © 2021 Fu et al. 2021 Fu et al. https://creativecommons.org/licenses/by/4.0/ This content is distributed under the terms of the Creative Commons Attribution 4.0 International license.

10.1128/mBio.00567-21.8 MOVIE S1 Time-lapse imaging showing cell enlargement of isolate F4 inside macrophages. Red arrows point to examples of cell enlargement at the end of the movie. Bar, 50 μm. Download Movie S1, AVI file, 19.9 MB. Copyright © 2021 Fu et al. 2021 Fu et al. https://creativecommons.org/licenses/by/4.0/ This content is distributed under the terms of the Creative Commons Attribution 4.0 International license.

10.1128/mBio.00567-21.9 MOVIE S2 Time-lapse imaging of macrophages containing Ftc555-1 as a control for the experiment shown in Movie S1. Bar, 50 μm. Download Movie S2, AVI file, 19.6 MB. Copyright © 2021 Fu et al. 2021 Fu et al. https://creativecommons.org/licenses/by/4.0/ This content is distributed under the terms of the Creative Commons Attribution 4.0 International license.

**FIG 8:**
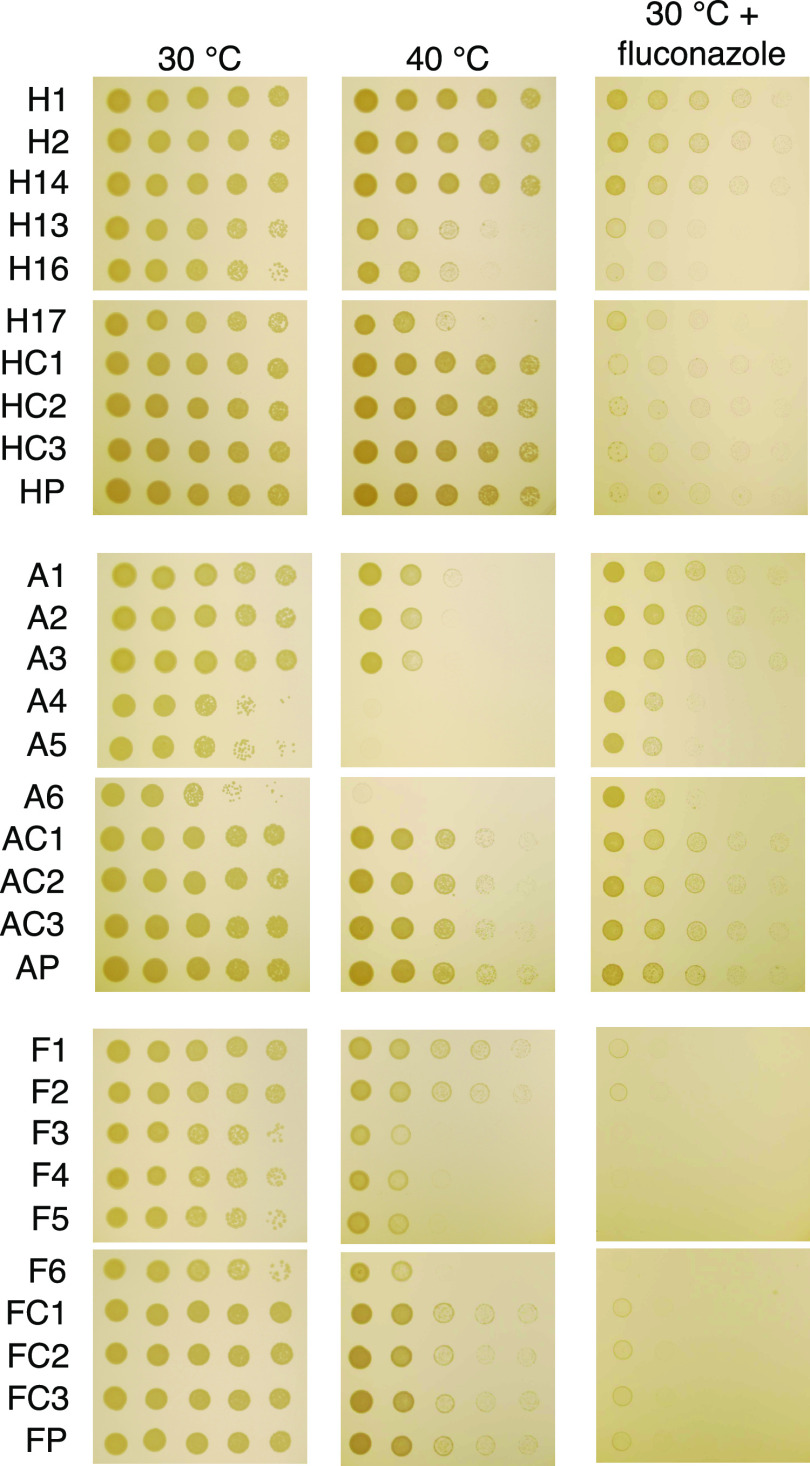
The growth of parent strains and isolates under stress conditions. Cells were 10-fold serially diluted and spotted onto Sabouraud medium with or without fluconazole (16 μg/ml) and grown for 2 days at 30°C or 40°C. Amoeba-passaged isolates are labeled with numbers preceded by the letters H, A, and F to indicate their origin from strains H99, A1-35-8, and Ftc555-1, respectively. HP, AP, FP: parental strains; HC, AC, FC: controls.

We further characterized the isolates under in stress conditions by analyzing their growth under thermal stress and exposure to the antifungal drug fluconazole (Fig. 8). Isolates H13, H16, and H17 had reduced growth at 40°C and in the presence of fluconazole, while H1, H2, and H14 had slightly increased their resistance to fluconazole compared to that of their ancestral strain. Isolates A4 to A6 and F3 to F6 displayed defects in growth at high temperature and after exposure to fluconazole.

Overall, the data show that the phenotypic changes were broad and diverse among isolates. A summary table with all isolates and their virulence factor phenotypes is provided in Fig. S2 in the supplemental material.

10.1128/mBio.00567-21.4 FIG S2 Summary of phenotypic changes that occurred in amoeba-passaged isolates. Download FIG S2, PDF file, 0.05 MB. Copyright © 2021 Fu et al. 2021 Fu et al. https://creativecommons.org/licenses/by/4.0/ This content is distributed under the terms of the Creative Commons Attribution 4.0 International license.

### Genomic analysis and sequencing results.

A prior study showed that DNA mutation was involved in pseudohyphal formation during amoeba interaction (55). To find out if there are any such mutations or other mutations in our experiments, the genomes of all isolates were sequenced. Single-nucleotide polymorphisms (SNPs) and indels were identified compared to the H99 reference genome (Table 1; see also Table S2 in the supplemental material). Genome sequencing revealed that H and A isolates acquired only small numbers of SNPs and indels during amoeba passage, whereas the rate of mutations in the Ftc555-1 isolates was 10 times higher, ranging from 22 to 77 SNPs (total of 225 SNPs) and 7 to 15 indels (total of 34 indels) in these isolates. Among those SNPs, three SNPs were annotated as high-impact mutations resulting in disruption of the coding region (early stop codons and splice site mutations) (Table 1). Nonsynonymous SNP changes and indels were identified in CNAG_03013 (*OPT1*), which encode an oligopeptide transporter, commonly in all three strain backgrounds. These SNPs contributes to the changes in the sequence of *OPT1*, which include a missense mutation resulting in the replacement of methionine 484 with arginine in H1, H2, and H14, a single-nucleotide deletion causing a frameshift at P358 in A1, a nonsense mutation in A2 and A3, and a splice site mutation in F1. Opt1 has been shown to be required for transporting Qsp1, a quorum-sensing peptide, into the receiving cells (56). Another SNP results in a nonsense mutation (G407*) in CNAG_00570, which encodes Pkr1 (AMP-dependent protein kinase regulator) in F5 and F6 isolates. In addition, F3 and F4 isolates carry a single-nucleotide deletion in CNAG_00570 that leads to a frameshift at residue 194 of 482. Pkr1 is one of the important components of cAMP/protein kinase A (PKA) pathway and negatively regulates Pka activity, which is involved in morphogenesis, nutrient acquisition, stress responses, and virulence in C. neoformans (57). An SNP found in H1, H2, and H14 isolates causes an intron variant in a gene encoding a protein kinase (CNAG_02531; *CPK2*) as part of the MAPK protein kinase family. Loss of CPK2 reduces melanin production in Niger seed medium (58). In A1, one missense SNP was found in CNAG_01101, which encodes a hypothetical protein with a centrosomin N-terminal motif. SNPs were also identified in A2 and A3 isolates, resulting in a missense mutation in CNAG_02858, which encodes adenylsuccinate synthetase. Another SNP in the A2 isolate was found in an intergenic region, a site with a high fraction of ambiguous calls. Isolates A4 to A6 had a single-nucleotide deletion at gene CNAG_03622 (*TAO3*), which led to a frameshift at residue 150 of 2,392. This mutation is consistent with the finding in a previous study that *TAO3* mutation led to the pseudohyphal phenotype (55). In summary, there are three noteworthy observations in the sequence data, as follows: (i) the gene CNAG_03013 (*OPT1*) was affected by nonsynonymous SNP changes in all three strain backgrounds, (ii) the previously described *TAO3* mutation responsible for pseudohyphal or hyphal formation was found in our isolates A4, A5, and A6 (55), and (iii) no SNPs or indels were found in some of the isolates, including H13, H16, and H17, suggesting that the phenotypic changes observed did not originate from single-nucleotide variants in the genome.

**TABLE 1:** High- and moderate-impact SNPs and indels found in passaged isolates

Gene identifier	Gene function	Isolates	Chromosome	Position	Reference	Alternate	Effect of mutation
CNAG_03013	Oligopeptide transporter	H1, H2, H14	3	211613	T	G	M484R
		A1	3	211137	GC	G	Frameshift at P358
		A2, A3	3	213165	G	A	Nonsense mutation W932^*b*^
		F1^*a*^	3	213566	G	T	Splice site mutation
CNAG_00570	cAMP-dependent protein kinase regulator	F5, F6^*a*^	1	1469244	C	A	Nonsense mutation G407^*b*^
		F3, F4^*b*^	1	1469985	GT	G	Frameshift at N194
CNAG_02531	Calcium-dependent protein kinase	H1, H2, H14	6	68953	C	A	Intron variant
CNAG_01101	Hypothetical protein	A1	5	1208219	T	C	R478G
CNAG_02858	Adenylsuccinate synthetase	A2, A3	3	594765	A	G	I346V
Intergenic region		A2	13	592173	C	T	
CNAG_03622	Cell polarity	A4, A5, A6	2	363200	CA	C	Frameshift at N150
CNAG_01506	Hypothetical protein	FC2^*a*^	11	136455	T	G	Splice site mutation

a Only high-impact mutations of passaged Ftc555-1 isolates are shown.

b Only selected high-impact indels of passaged Ftc555-1 isolates are shown here. Others are shown in Table S2 in the supplemental material.

10.1128/mBio.00567-21.2 TABLE S2 High-impact indels found in passaged Ftc555-1 isolates. Download Table S2, DOCX file, 0.02 MB. Copyright © 2021 Fu et al. 2021 Fu et al. https://creativecommons.org/licenses/by/4.0/ This content is distributed under the terms of the Creative Commons Attribution 4.0 International license.

To determine if the high-impact mutations we identified in genes *PKR1*, *OPT1*, CNAG_02531, and CNAG_01506 are responsible for resistance to the killing of amoebae, deletion mutants of the candidate genes in the H99 background were coincubated with amoebae on solid medium. However, the predation zones from these mutants were comparable with those of the parental strain (see Fig. S3 in the supplemental material).

10.1128/mBio.00567-21.5 FIG S3 Amoebae killing assay on Cryptococcus neoformans deletion mutants. Mutants showed comparable predation zones to those of their parental strains. Download FIG S3, PDF file, 0.04 MB. Copyright © 2021 Fu et al. 2021 Fu et al. https://creativecommons.org/licenses/by/4.0/ This content is distributed under the terms of the Creative Commons Attribution 4.0 International license.

### Aneuploidy.

**FIG 9:**
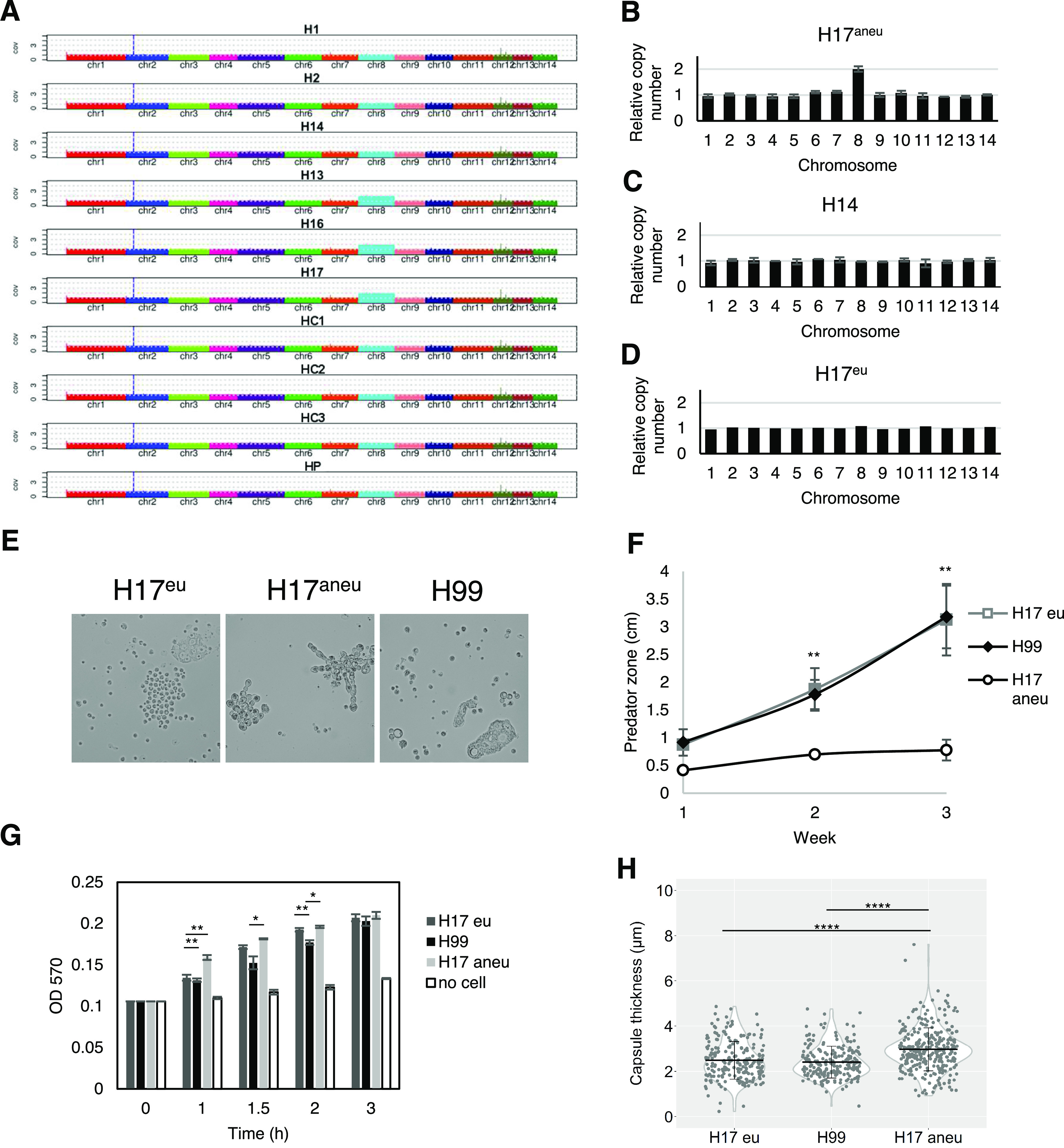
Aneuploidy plays a role in pseudohyphal formation. (A) Chromosomal copy numbers of H99 isolates were determined based on depth of sequence coverage normalized by the average genome-wide sequence depth. (B) The relative chromosome copy number of isolate H17 was obtained by quantitative PCR (qPCR). H17 has duplication of chromosome 8. (C) No aneuploidy was found in isolate H14. (D) Chromosome duplication in H17 is eliminated by passaging H17 in fresh Sabouraud medium for 30 days. (E) The H17 euploid (H17^Eu^) strain did not form pseudohyphae as rapidly as the H17 aneuploid strain. (F) The H17^Eu^ euploid strain has larger predation zone than that of H17^Aneu^. Data represent the mean of three biological replicates per biological sample, and error bars are SD. **, *P < *0.01, compared to H17^Aneu^ by one-way ANOVA and followed by Tukey’s multiple-comparison test. (G) The H17^Eu^ strain has lower urease activity than that of H17^Aneu^ and comparable urease activity to that of H99 at an early time point (1 h). Data represent the mean of two biological replicates per biological sample, and error bars are SD. (H) H17^Eu^ has smaller capsule size than that of H17^Aneu^, but a similar capsule size to that of H99.

We next hypothesized that emergence of aneuploidy could be a source of evolutionary adaptation because aneuploidies are frequent in C. neoformans and have been shown to play crucial roles in stress resistance (59, 60). To this end, the chromosomal copy numbers of the isolates evolved from all three strain backgrounds were defined based on the normalized depth of sequence coverage. The analysis revealed that there were duplications of chromosome 8 in isolates H13, H16, and H17, but no chromosomal duplication has been found in other isolates (Fig. 9A). The results were confirmed by quantitative (qPCR) with two selected isolates, H14 and H17 (Fig. 9B and C). We next investigated if this chromosomal duplication was responsible for the pseudohyphal formation and other phenotypic changes. In order to do so, H17 was passaged in fresh rich medium every day for 30 days to eliminate the duplication. The elimination was confirmed by qPCR (Fig. 9D). The H17 euploid strain (H17^eu^) was then coincubated with amoeba culture in solid medium, and samples were taken from the edge of the predation zone and visualized under a microscope. No pseudohyphae could be observed in H17^eu^ (Fig. 9E). In this case, the observation was similar to what we found in H99, but distinct from that in the H17 aneuploid strain (H17^aneu^), which primarily formed pseudohyphae after 1 week of coincubation (Fig. 9E). Not surprisingly, H17^eu^ had a decreased ability for amoeba resistance, having a similar size of the predation zone to that of H99, while H17^aneu^ had a smaller predation zone (Fig. 9F). The capsule size of H17^eu^ was smaller than that of H17^aneu^ and was similar to that of H99, suggesting that the duplication of chromosome 8 results in a larger capsule size (Fig. 9G). After 1 h, H17^eu^ had lower urease activity than that of H17^aneu^ but a comparable level to that of H99 (Fig. 9H). However, the urease activity of H17^eu^ increased more quickly than that of H99 after 1.5 h. The result implied that the chromosomal duplication may be responsible in part for the high urease activity found in H17^aneu^.

**FIG 10:**
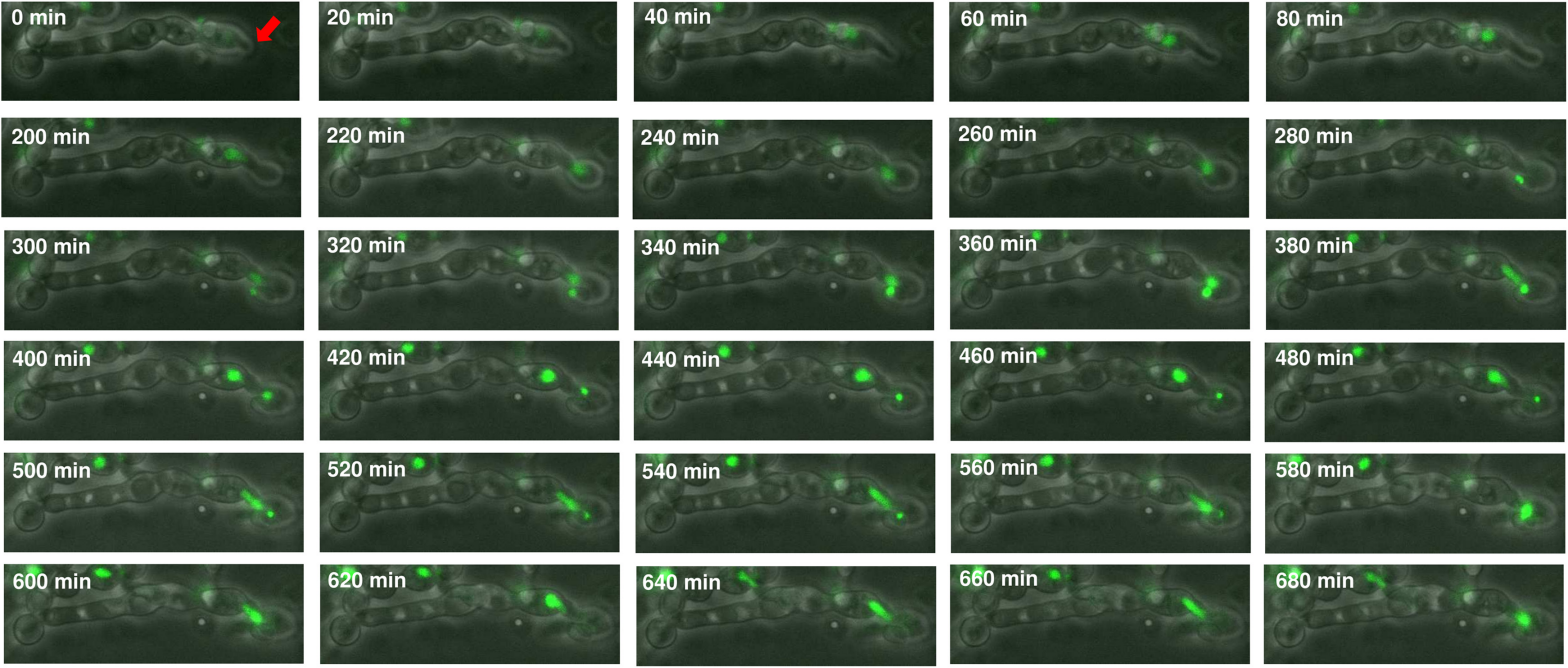
Time-lapse imaging showing nuclear division of pseudohyphae. The images of pseudohyphae of amoeba-passaged H99 GFP-H2B strain were taken by phase-contrast and fluorescence microscopy. Buds (red arrow) formed between 0 and 220 min. The nuclei migrated into the daughter cells at 240 min and separated at 300 min. Nuclear division was completed at 400 min. However, the nuclei from mother cells reentered the daughter cells at 500 min and underwent fusion at 580 min.

Aneuploidy can arise from a multinucleate state through transient polyploidization after failed cytokinesis or cell fusion. The filamentous multinucleate fungus Ashbya gossypii frequently exhibits both polyploidy and aneuploidy after cell division (61). Since pseudohyphae have a cytokinesis defect and multiple nuclei within a common cytosol, we asked if the pseudohyphal formation might lead to ploidy variation and thus could become one of the sources of phenotypic variation. Consequently, H99 cells expressing green fluorescent protein-labeled histione-2 (GFP-H2B) that were passaged through amoebae were visualized by time-lapse imaging, and a nucleus fusion was observed in one of the pseudohyphae after nuclei separation (Fig. 10 and Movie S1). This event provides evidence that polyploidization can exist in pseudohyphae and thus may have a high chance of leading to aneuploidy and phenotypic variation.

### Effects of amoeba selection on interactions with murine macrophages.

**FIG 11:**
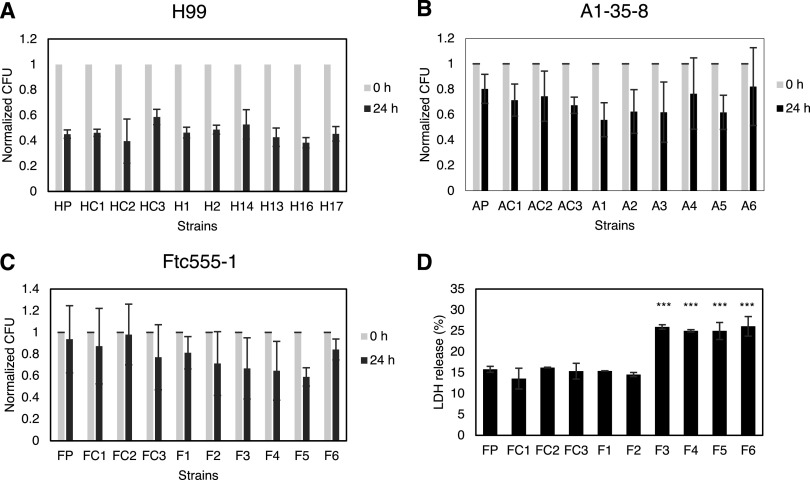
(A to C) The survival of parent strains and isolates in culture with BMDMs. The survival of (A) H99, (B) A1-35-8, and (C) Ftc555-1 isolates was determined by CFU after 0 and 24 h phagocytosis. The percentage of survival was calculated by normalizing the CFU value of 24 h infection to time zero. HP, AP, FP: parental strains; HC, AC, FC: controls Data represent the mean of three biological replicates, and error bars are SD. There was no statistically significant difference between isolates and their parental strains as determined by one-way ANOVA followed by Tukey’s multiple-comparison test. (D) BMDMs were infected with Ftc555-1 isolates for 48 h. Lactate dehydrogenase (LDH) release from damaged BMDMs into culture supernatant was assayed. ***, *P < *0.001; one-way ANOVA followed by Tukey’s multiple-comparison test.

Based on the changes of multiple virulence-related phenotypes, we expected that some of the isolates would have a better survival when interacting with macrophages. However, there was no significant change in intracellular survival among all the isolates (Fig. 11A to C). Nevertheless, we cannot rule out the possibility that isolates may cause damage to macrophages. Since isolates F3 to F6 underwent cell enlargement inside macrophages, we hypothesize that the increased cell size may physically rupture macrophages. Therefore, we measured the release of lactate dehydrogenase (LDH) from the macrophages when they were infected with Ftc555-1 isolates. Indeed, it was found that LDH release was significantly induced from the macrophages containing F3 to F6 compared to that from those containing the ancestral strain (Fig. 11D), suggesting that F3 to F6 and their enlarged yeast cells cause damage to their host cells. While it may seem paradoxical that incubation of F isolates with macrophages resulted in no major changes in CFU of F isolates while increasing phagocytic cell damage, we note that these can be independent effects. For example, differences in cryptococcal cell proliferation rate could change CFU, whereas cell enlargement, which can disrupt the phagosome through physical stress (62), can be associated with elongated cell cycle phases (63).

### Virulence in mice and moth larvae.

**FIG 12:**
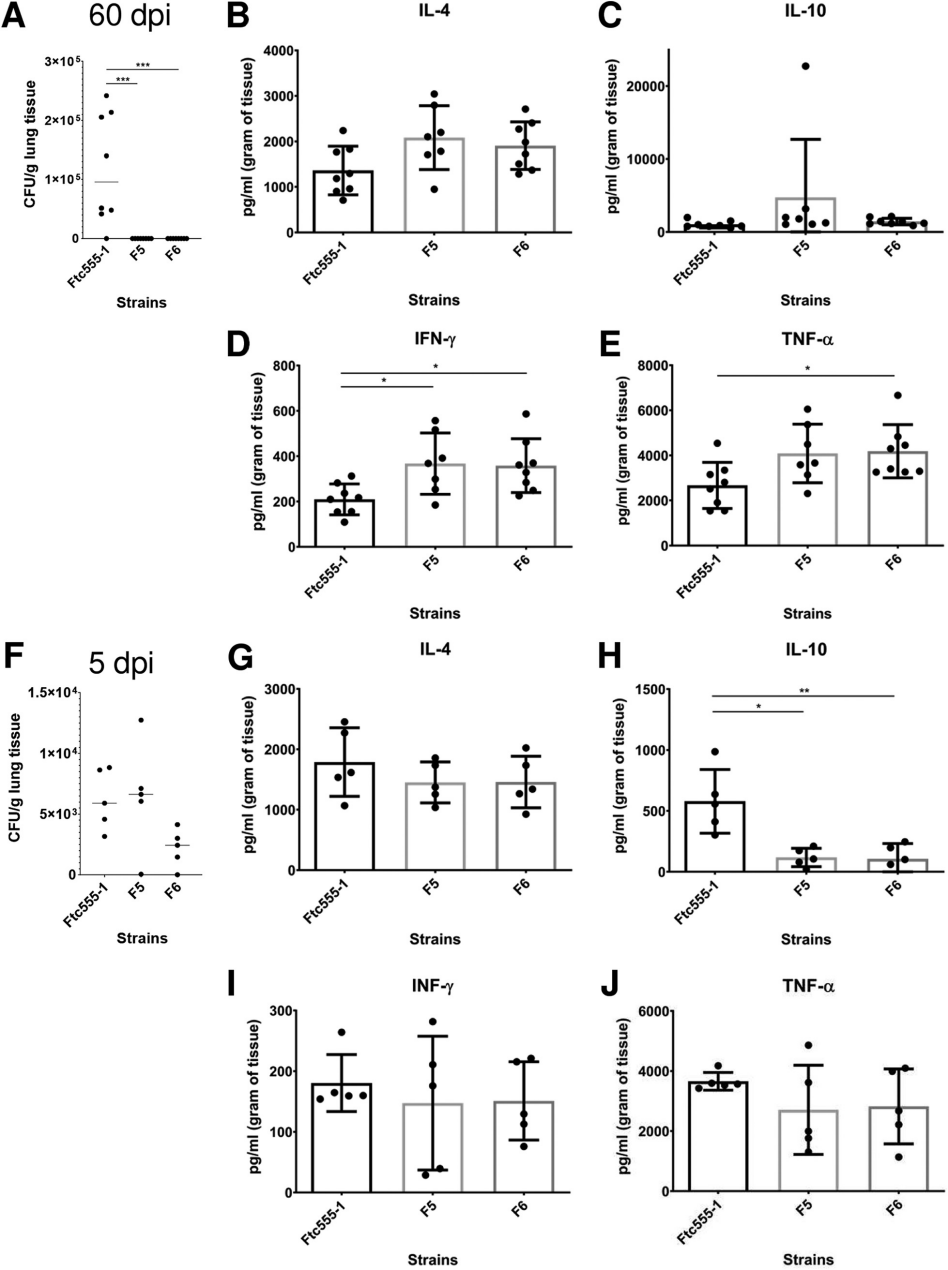
Fungal burden and cytokine production in the lung after infection with Ftc555-1, as well as its isolates F5 and F6. After 60 days of infection, mice were sacrificed, and fungal burden in the lung was determined by CFU counting (A). The levels of cytokines (B) interleukin 4 (IL-4), (C) IL-10, (D) interferon gamma (IFN-γ), and (E) tumor necrosis factor alpha (TNF-α) in the lung were measured by enzyme-limited immunosorbent assay (ELISA). At 5 days postinfection, (F) fungal burden and the amounts of cytokines (G) IL-4, (H) IL-10, (I) IFN-γ, and (J) TNF-α in the lung were also measured. All data represent the mean of eight mice per group, and errors bars are SD. One-way ANOVA with Kruskal-Wallis nonparametric test was used and followed by Bonferroni’s multiple-comparison test. *, *P < *0.1; **, *P < *0.01; ***, *P < *0.001.

The deletion of PKR1 was reported to be hypervirulent in mice (64). Since isolates F5 and F6 were more cytotoxic to macrophages and contained loss of function mutations in *PKR1*, we investigated their virulence and their parental strain Ftc555-1 in a murine infection model. However, all animals survived for 60 days after intranasal inoculation (data not shown). Lung fungal burden was determined by enumerating CFU. Only the cells of the initial isolate (Ftc555-1) were detected in the mouse lung after this incubation period, and there was considerable mouse-to-mouse variation in CFU. Hence, the two isolates carrying *PKR1* mutations were cleared from the lungs 60 days after inoculation (Fig. 12). Consequently, we explored early times of infection and noted that at day 5 after challenge, both Ftc555-1 and F5 had comparable fungal burdens, while that of F6 was reduced (Fig. 12). Hence, all three strains were able to establish themselves in mice initially and avoid clearance by innate immunity, but the F5 and F6 strains were subsequently cleared, presumably by the development of acquired immunity, which normally occurs at a later stage of the infection.

To gain more insight into the immune responses elicited by Ftc555-1, F5, and F6 in the lung, we studied several cytokine responses. In the lungs, the levels of proinflammatory cytokines, including tumor necrosis factor alpha (TNF-α) and interferon gamma (IFN-γ), were increased and remained high after 60 days postinfection with F5 and F6 strains (Fig. 12). F5 and F6 elicited lower lung levels than Ftc555-1 of interleukin 10 (IL-10), which could help their clearance from lung tissue relative to that of Ftc555-1, since reduction of this anti-inflammatory cytokine is associated with increased resistance to cryptococcal infection in mice (65). Interestingly, the levels of the same molecules were different when we analyzed the systemic immune response as measured by cytokines in their spleens (see Fig. S4 in the supplemental material). These results suggest that eliciting high levels of these cytokines may stimulate an inflammatory reaction, which could be associated with resolution of the infection of the F5 and F6 strains. These cytokine results show that F5 and F6 strains elicited quantitatively different immune responses from the parent strain Ftc555-1, consistent with the notion that the differences in virulence observed for these strains reflect differences in the effectiveness of the immune responses triggered.

10.1128/mBio.00567-21.6 FIG S4 Cytokine production in mouse spleen after infection with Ftc555-1, as well as with its isolates F5 and F6. After 60 and 5 days of infection, mice were sacrificed, and the level of cytokines (A, E), interleukin 4 (IL-4), (B, F) IL-10 (D), interferon gamma (IFN-γ), and (D, G) tumor necrosis factor alpha (TNF-α) in the lung were measured by enzyme-limited immunosorbent assay (ELISA). All data represent the mean of eight mice per group, and errors bars indicate standard deviation (SD). For determination of cytokine levels, one-way analysis of variance (ANOVA) with a Kruskal-Wallis nonparametric test was used and followed by Bonferroni’s multiple-comparison test. A *t* test was used to compare the number of CFU for different groups. Download FIG S4, PDF file, 0.2 MB. Copyright © 2021 Fu et al. 2021 Fu et al. https://creativecommons.org/licenses/by/4.0/ This content is distributed under the terms of the Creative Commons Attribution 4.0 International license.

We also examined the virulence of the isolates using a wax moth larval model, and isolates H13, H16, and H17 were less virulent than their parental strain (see Fig. S5A in the supplemental material). This may be due to the fact that these isolates can rapidly form pseudohyphae in the larvae, and pseudohyphal C. neoformans cells are attenuated for virulence in wax moth larvae (55).

10.1128/mBio.00567-21.7 FIG S5 Virulence of parents and variant isolates in the Galleria mellonella larvae infection model. The Kaplan-Meier plots shows the survival of G. mellonella after injection of cryptococcal cells (10^3^ cells/larva). Download FIG S5, PDF file, 0.1 MB. Copyright © 2021 Fu et al. 2021 Fu et al. https://creativecommons.org/licenses/by/4.0/ This content is distributed under the terms of the Creative Commons Attribution 4.0 International license.

## DISCUSSION

In the past 2 decades, the concept that amoebae act as a selective pressure for virulence traits of environmental microbes has gained considerable traction. For fungal pathogens, concordance between virulence factor function in amoebae and macrophages has been demonstrated for C. neoformans (15, 40), Aspergillus fumigatus (13, 66), and Paracoccidioides spp. (24), but many questions remain regarding how fungal-protozoal interactions select for mammalian virulence. In this study, we investigated how interactions with amoebae affected the phenotype and genotype of C. neoformans to explore the mechanisms behind this long-term evolutionary adaptation. Our results provide new insights on how amoeba predation can drive the evolution of C. neoformans, since survivors emerge that show major phenotypic and genetic differences from the founder strain. This phenotypic diversity may facilitate C. neoformans adaptation to different hosts and alter its virulence.

Pseudohyphal formation was the most common response to C. neoformans survival when faced with amoeba predation. This result confirms an older observation that pseudohyphal formation was an “escape hatch” for C. neoformans survival when preyed upon by amoebae (67). Different fungal morphologies are reported to trigger different killing mechanisms by amoebae (68), and the C. neoformans filamentous form may be more resistant to killing. Similarly to our observation, Nielson et al. (67) reported that when C. neoformans was cocultured with amoebae, most of the fungal cells were killed, with survivors forming colonies that contained pseudohyphae. Most of their isolates remained pseudohyphal, with only one out of eight isolates reverting back to the yeast form. That result differed from ours, since most of the pseudohyphal isolates in this study reverted to yeast forms after removal from the amoeba culture, such that only 3 of 18 isolates studied in detail maintained a stable pseudohyphal phenotype. Those three isolates (A4 to A6) have a single-nucleotide deletion in the *TAO3* gene, shown by whole-genome sequencing, which is consistent with mutations in the RAM/MOR (regulation of Ace2 and morphogenesis/morphogenesis-related NDR kinase) pathway of the pseudohyphal variants reported in a previous study (55).

Previous studies have focused primarily on cryptococcal isolates with pseudohyphal phenotypes derived from amoeba, but in this study, we investigated in detail those amoeba-resistant isolates with unstable pseudohyphal phenotypes. We found that although some of the isolates (H13, H16, and H17) reverted to yeast, they were able to form pseudohyphae more quickly than their parental strain when they were exposed to amoebae again. These isolates were less virulent in a Galleria infection model, a finding consistent with prior reports that the pseudohyphal strains were less virulent in animal models. Interaction with amoebae also resulted in measurable virulence-related phenotypic changes in C. neoformans, confirming that amoebae can play a powerful role in the selection of virulence factors, which are related to the pathogenesis of human disease. It is of note that we selected only six isolates from each strain for further characterization, but all of them had changes, suggesting that the microevolution occurs frequently and rapidly when isolates are exposed to amoebae. Moreover, the changes were pleiotropic and included differences in colony morphology, capsule size, cell size, urease activity, melanin production, and susceptibility to thermal stress and an antifungal drug. However, isolates studied revealed a different configuration of phenotypic changes, although they tended to cluster in groups from the same surviving pseudohyphal colony (see Fig. S1 in the supplemental material). Overall, the interaction of C. neoformans with amoeba-passaged isolates increased phenotypic diversity. Since there are many types of amoeboid predators in the soil and Cryptococcus species do not know the identity of the phagocytic predator, generating great diversity in strains could provide this fungus with insurance that some will survive. Hence, the diversity observed among isolates that survived amoeba predation suggests a bet-hedging strategy for survival based on the generation of phenotypic diversity.

To identify the mechanism for the phenotypic changes, we compared the whole-genome sequencing of isolates and ancestral strains using deep sequencing to identify point mutations, amplification or deletion of chromosomal segments, and whole-chromosome aneuploidy. We found that there were only two SNPs in H99-derived isolates and four SNPs and two indels in the A1-35-8-derived isolates. Isolates from the same surviving pseudohyphal colonies had similar SNPs, which is consistent with the similarity of their phenotypic changes, suggesting that the point mutations may be associated with some of the phenotypic changes. Interestingly, there were in total 252 SNPs in Ftc555-1-derived isolates, with an average of 48 SNPs among isolates (range of 22 to 80), a rate approximately10 times higher than those for H99 and A1-35-8. That may be explained by the fact that the ancestral Ftc555-1 strain contains a splice donor site mutation in *MLH1*, a gene involved in mismatch repair of nuclear DNA. This predicted high-impact loss-of-function mutation (G-to-A change at position 1270268 of chromosome 6) is also found in all sequenced Ftc555-1 progeny isolates. Since the Idnurm laboratory has previously reported that the loss of *MLH1* results in elevated mutation rates (69), Ftc555-1 is likely to be a hypermutator strain. Increased mutation rates will drive phenotypic variations, and some of those may be adaptive for survival in stressful environments, leading to rapid microevolution. On the other hand, the sequencing revealed that one gene (CNAG_03013; *OPT1*) was impacted by nonsynonymous SNP changes and single-nucleotide deletion in all three strain backgrounds. *OPT1* has been identified by the Madhani group as an oligopeptide transporter required for transporting Qsp1, a quorum-sensing peptide, into the receiving cells (56). Deletion of *OPT1* produces phenotypes similar to those of our isolates, including increased capsule size and reduced melanin production, suggesting that this mutation may cause some of the phenotypic changes in our isolates. Increased capsule size can protect C. neoformans against amoeba phagocytosis (46). Moreover, amoebae are known to produce antimicrobial pore-forming peptides (70), and it is conceivable that mutation of *OPT1* could reduce their importance and protect C. neoformans. By reviewing the published sequences of 387 clinical and environments strains (71), we found that 6 of 287 clinical isolates had high-impact potential loss-of-function mutations in *OPT1*, but there were no *OPT1* mutations in the 100 environmental isolates. The Fraser laboratory also reported that one of the clinical isolates in their study contains an inversion in chromosome 3 that affect two genes, one of them being *OPT1* (72). The relatively high frequency of mutations in *OPT1* among clinical isolates suggests that this gene may be under particular selection during human infection. Another interesting mutation found in Ftc555-1 isolates was in the gene *PKR1*. This was a high-impact mutation in F3, F4, F5, and F6, which exhibited phenotypes of titan cells and enlarged capsules inside macrophages and in macrophage medium. Pkr1 is known to be a negative regulator of titan cell formation and capsule enlargement in laboratory strains and clinical isolates (64, 73). A *pkr1* deletion mutant exhibits both enlarged capsule and titan cell production. It is also hypervirulent in a murine infection model (64).

The relatively small number of SNPs raises the question of how some of these strains changed rapidly in response to amoeba predation, resulting in broad and rapid phenotypic changes. Therefore, we also investigated the impact of whole-chromosome aneuploidy on isolates. An extra copy of chromosome 8 has been found in three isolates (H13, H16, and H17) which were isolated from the same surviving pseudohyphal colony. Aneuploidy is caused by abnormal chromosomal segregation and can happen within even a single mitotic division, so this type of mutant can occur rapidly. This drastic DNA structural change often results in decreased fitness (74). However, when fungi are exposed to stressors, such as antifungal drugs, specific chromosomal aneuploidies can be advantageous through selection for increased gene expression of a subset of genes (60, 75–79). In Candida albicans and C. neoformans, extra copies of specific chromosomes containing drug resistance genes have been frequently found in antifungal drug resistance strains (60, 77, 78). Likewise, C. neoformans could gain an extra chromosome as a solution for adaptation when the fungi encounter threats from amoebae. For instance, chromosome 8 contains one gene (*ZNF2*) that encodes a zinc finger transcription factor that drives hyphal growth upon overexpression (80). Chromosome 8 also contains another gene (*CBK1*) that is responsible for pseudohyphal formation (55, 81). *CBK1* encodes a serine/threonine protein kinase, which is one of the components of the RAM pathway. Mutants in the RAM pathway have a pseudohyphal phenotype, but we are not aware of any reports showing the effect of the overexpression of *CBK1* on pseudohyphal morphology. Since filamentous morphologies are important for resistance to phagocytosis by amoebae, it is possible that duplication of chromosome 8 could rapidly increase cryptococcal fitness after exposure to amoebae. Indeed, when we reintroduced those aneuploid strains to amoebae, they could switch to filamentous forms more quickly than their parental strain and efficiently resisted killing by amoebae. When we eliminated the chromosomal duplication, the phenotypes were restored back to the wild-type level, supporting a strong link between duplication of chromosome 8, amoeba resistance and other changes in virulence phenotypes such as capsule size and urease activity. In addition, there are no point mutations or structural changes, such as amplification or deletion of chromosomal segments, in these isolates. Therefore, aneuploidy may be the major source of the phenotypic change in that particular group of isolates. However, aneuploidy was not found in isolates from other two strain backgrounds (A1-35-8 and Ftc555-1), so it may not be a general resistance trait.

Pseudohyphae are chains of elongated yeast cells that are unable to undergo complete cytokinesis, leading to multiple nuclei. Multinucleated cells showed a high level of chromosome instability, resulting in polyploidy and aneuploidy in eukaryotic cells (61). Previous study of live-cell imaging on Candida albicans showed that hyphal cells occasionally generated multinucleated yeast cells (82) with polyploidy and/or aneuploidy but there are very limited studies on whether pseudohyphal or hyphal formation may directly affect the ploidy variation. In this study, nuclear division, detected with GFP-H2B, was observed in cryptococcal pseudohyphae isolated from amoeba culture. Time-lapse imaging detected a nuclear fusion event, suggesting the cell experienced atypical nuclear division and may potentially undergo polyploidization, which frequently generates offspring with amplification of chromosomal segments or whole-chromosome aneuploidy. This result implies that interaction with amoebae not only contributes to the selection and maintenance of traits in C. neoformans, but also may drive heritable variation through pseudohyphae formation. However, a single event was observed, so this may not be a common escape strategy of C. neoformans.

The “amoeboid predator-fungal animal virulence hypothesis” posits that the capacity for virulence in soil fungi with no need for an animal host arose accidently from the selection of traits that promote survival against ameboid predators, which also function as virulence factors for animal infection (12). Consistent with this notion, there is a remarkable concordance between fungal phenotypes that promote survival against amoebae and in animal hosts (13, 15), and passage in amoeba is associated with increased virulence for several fungal species (24, 39, 83). Analysis of virulence for the amoeba-selected strains described in our study in wax moths revealed no major changes in virulence from the parental strains. It is possible that this host does not discriminate between passaged and nonpassaged C. neoformans cells, or that none of the isolates tested gained or lost traits associated with virulence in that particular host. It is also possible that these strains already had the maximum pathogenic potential (84) for these animal hosts, which could not be further increased by amoeba interactions. However, we did observe that some amoeba-passaged strains were significantly more cytotoxic for macrophages *in vitro*. This result is consistent with the finding that those strains also had great resistance to amoeba killing. The mechanism behind that is still unclear. However, those particular amoeba-passaged strains can form larger-sized cells and capsules in both amoeba and macrophage culture, which may help them escape from, and damage host cells. These results fit the hypothesis that amoebae are the training grounds for macrophage resistance of pathogens, since the hostile environments in amoebae and macrophages are similar. Among these strains, the virulence of isolates F5 and F6 were further tested in a murine infection model. These particular strains were picked because they acquired a mutation in *PKR1*, and deletion of *PKR1* has been shown to increase virulence (64). However, neither F5 and F6 exhibited a hypervirulence phenotype during murine infection, and instead were cleared faster than their parental isolate. It is noteworthy that the nonsense mutation found in F5 and F6 is located in codon 407, which is only 75 codons prior to the original stop codon of *PKR1*. It is possible that the mutation results in altered function rather than loss of function and that this is not sufficient to reproduce the hypovirulence phenotype caused by full *PKR1* knockout. Microbial virulence is a complex property that is expressed only in a susceptible host, and host damage can come from the microbe or the immune response (85). Both F5 and F6 were able to establish themselves in the lung but triggered a more effective immune responses that cleared them. This finding implies the occurrence of other amoeba-selected changes that affect the immune response, including overriding of the hypervirulence phenotype caused from the mutation of *PKR1* by compensation from other mutations or changes.

The amoeba-passaged C. neoformans isolates selected in our study differ from those reported in prior studies (24, 39, 83) in that they did not increase in virulence. Instead, we observed reductions in murine virulence from their long interaction with amoeba for two of the isolates studied, despite increased capacity to damage macrophages. Given the pleiotropic changes observed in our isolate set, it is possible that we did not sample sufficient numbers to observe more virulent strains. Our study also differs from prior amoeba-C. neoformans studies (39) in that it involved prolonged selection on a semisolid agar surface under conditions that favored amoeba by the presence of cations. Under these conditions, amoeba dominance is manifested by a zone of fungal growth clearance where only occasional C. neoformans colonies emerged after several weeks. These colonies presumably emerged from resistant cells that survived the initial amoeba onslaught and gave rise to the variant strains that were analyzed in this study. We suggest that these amoeba-resistant cells were very rare in the initial parental population and emerged from the mechanisms discussed above, namely, mutation and aneuploidy, which by chance conferred amoeba resistance upon those cells. Alternatively, these colonies represent rare cells that were able to sense the amoeba danger and turn on diversity-generating mechanisms that occasionally produced amoeba-resistant strains. In this regard, C. neoformans can sense amoebae and respond by increasing the size of its capsule by sensing protozoal phospholipids (40), but this process takes time, and fungal cell survival probably depends on the race between adaptation and predation. The selection versus adaptation explanations for the origin of these are not mutually exclusive, and both could have been operational in these experiments. These survivor cells then grew into a colony under constant amoeba selection where they gave rise to progeny cells where these phenotypic diversity-generating mechanisms were maintained and amplified, thus accounting for the phenotypic diversity observed in this study.

In summary, amoeba predation places selective pressure in C. neoformans, resulting in the rapid emergence of new phenotypes associated with mutations and aneuploidy, which combine to create great phenotypic diversity. The effect of phenotype diversification on the fitness of the fungi differs within the same or different hosts, suggesting a bet-hedging strategy by C. neoformans that spreads the risk in situations where the environmental threat is unpredictable. Given that human infection also results in rapid fungal microevolution in this host (86, 87), it is possible that similar mechanisms occur *in vivo* when this fungus comes under attack by immune cells. Indeed, macrophages appear to also use a bet-hedging strategy in phagosomal acidification to control microbes (88), and several studies have shown microevolution of *Cryptococcus* during mammalian infection (72, 87, 89, 90). A bet-hedging strategy that generates a prodigious number of phenotypes would increase survival in the face of unknown threats and could represent a general mechanism for survival in soils. Interference with the mechanism responsible for generating this plasticity could in turn result in new antimicrobial strategies that would reduce the emergence of diversity and thus simplify the problem for the immune response. Hence, it is interesting to hypothesize that amoeba predation in C. neoformans pushes a trigger that sets forth a series of events that generate diversity and that similar mechanisms exist in other soil fungi that must routinely confront similar stresses.

## MATERIALS AND METHODS

### Ethics statement.

All animal procedures were performed with prior approval from Johns Hopkins University (JHU) Animal Care and Use Committee (IACUC), under approved protocol number MO18H152. Mice were handled and euthanized with CO_2_ in an appropriate chamber followed by thoracotomy as a secondary means of death in accordance with guidelines on Euthanasia of the American Veterinary Medical Association. JHU is accredited by AAALAC International, in compliance with Animal Welfare Act regulations and Public Health Service (PHS) policy, and has a PHS Approved Animal Welfare Assurance with the NIH Office of Laboratory Animal Welfare. The JHU Animal Welfare assurance number is D16-00173 (A3272-01). JHU utilizes U.S. Government laws and policies for the utilization and care of vertebrate animals used in testing, research, and training guidelines for appropriate animal use in a research and teaching setting. Mice were maintained in a 12-h:12-h light-dark (LD) cycle and at constant temperature (22°C ± 1°C). They were allowed to free access to water and food. Mice were kept under these conditions for 1 week before and during the experiments.

### Cell culture.

Acanthamoeba castellanii strain 30234 was obtained from the American Type Culture Collection (ATCC). Cultures were maintained in yeast-peptone-glucose (YPG) broth (ATCC medium 712) at 25°C according to instructions from ATCC. C. neoformans var. *grubii* serotype A strain H99 and two environmental isolates, A1-35-8 and Ftc555-1, were used for the interaction with amoebae. The histone 2B-GFP-tagged (C1746) H99 strain that was used for visualization of nuclear division of pseudohyphae was obtained from Kyung Kwon-Chung (Bethesda, MD) (91). Cryptococcal cells were cultivated in Sabouraud dextrose broth with shaking (120 rpm) at 30°C overnight (16 h) prior to use in all experiments.

Bone marrow-derived macrophages (BMDMs) were isolated from the marrow of hind leg bones of 5- to 8-week-old C57BL/6J female mice (Jackson Laboratory, Bar Harbor, ME). For differentiation, cells were seeded in 100-mm tissue culture (TC)-treated cell culture dishes (Corning, Corning, NY) in Dulbecco’s modified Eagle medium (DMEM; Corning) with 20% L-929 cell-conditioned medium, 10% fetal bovine serum (FBS; Atlanta Biologicals, Flowery Branch, GA), 2 mM Glutamax (Gibco, Gaithersburg MD), 1% nonessential amino acids (Cellgro, Manassas, VA), 1% HEPES buffer (Corning), 1% penicillin-streptomycin (Corning), and 0.1% 2-mercaptoethanol (Gibco) for 6 to 7 days at 37°C with 9.5% CO_2_. Fresh medium (3 ml) was supplemented on day 3, and the medium were replaced on day 6. Differentiated BMDMs were used for experiments within 5 days after completed differentiation.

### Assay of A. castellanii and C. neoformans interaction.

Two hundred C. neoformans yeast cells were spread on Sabouraud agar and incubated at 30°C overnight. A. castellanii cells (5 × 10^3^) were dropped randomly at several locations on the agar plate containing C. neoformans. Plates were sealed with parafilm and incubated at 25°C for 3 to 4 weeks until surviving colonies of C. neoformans emerged.

To isolate an individual cell (in this case, hyphae or pseudohyphae) from the colony (Fig. 1D), surviving colonies were randomly picked from the plate to a 3-cm culture dish with phosphate-buffered saline (PBS) using pipette tips. Individual cells were picked under a light microscope using a pipette and transferred into fresh Sabouraud agar. The plates were incubated at 30°C. After 24 h of incubation, the morphologies of microcolonies were visualized using a Zeiss Axiovert 200M inverted microscope with a 10× phase objective. After 72 h of incubation, colony morphologies were examined using Olympus SZX9 microscope with a 1× objective and a 32× zoom range. Morphologies of cells from colonies were visualized using an Olympus AX70 microscope with a 20× objective using the QCapture Suite v2.46 software (QImaging, Surrey, Canada).

### Amoebae killing assay.

C. neoformans cells (5 × 10^6^ cells) were spread as a cross onto Sabouraud agar and incubated at 30°C for overnight. A. castellanii cells (10^4^) were dropped at the center of the C. neoformans cross. The plates were sealed in parafilm and incubated at 25°C. The distance from center to the edge of the clear predation zones in four directions was measured after 1 to 3 weeks incubation. Data are represented as the average of the distances of the clear zone from four direction.

C. neoformans cells were also taken from the edge of the clear zone and at the end of the cross after 1 week of incubation and visualized using an Olympus AX70 microscope with 20× objective. For samples of Ftc555-1 strains, the cells were counterstained with India ink.

### Capsule and cell size.

C. neoformans cells were incubated in minimal medium (15 mM dextrose, 10 mM MgSO_4_, 29.4 mM KH_2_PO_4_, 13 mM glycine, and 3 μM thiamine-HCl) at 30°C for 72 h. In addition, Ftc555-1 and its isolates were incubated in medium for BMDMs at 37°C for 24 h. BMDMs (1.5 × 10^6^ cells) were also infected with Ftc555-1 and its isolates (1.5 × 10^6^ cells) in 6-well plates. After 24 h of infection, the culture supernatant was collected, and the plates were washed once to collect the extracellular C. neoformans. Intracellular C. neoformans was collected by lysing the host cell with sterile water. The cells were stained with 0.1% Uvitex 2B (Polysciences, Warrington, PA) for 10 min and washed two times with PBS. The capsule was visualized by India ink negative staining by mixing cell samples with equal volumes of India ink on glass slides and spreading the smear evenly with coverslips. Images with a minimum of 100 randomly chosen cells were taken using an Olympus AX70 microscope with 40× objective using bright-field illumination and the 4′,6-diamidino-2-phenylindole (DAPI) channel. The areas of the cell body and the whole cell (cell body plus capsule) were measured using ImageJ software. Capsule thickness was calculated by subtracting the diameter of the whole cell from that of the cell body. Cell size is presented as the diameter of the cell body without the capsule. Three biological independent experiments were performed for each sample.

### Lactate dehydrogenase release assay.

BMDM cells (5 × 10^4^ cells/well) were seeded in 96-well plates with BMDM medium for overnight. To initiate the phagocytosis, BMDMs was infected with C. neoformans (5 × 10^5^ cells) opsonized with 18B7 monoclonal antibody (MAb), which binds capsular glucuronoxylomannan, at a concentration of 10 μg/ml. The culture plates were centrifuged at 1,200 rpm for 1 min to settle yeast cells on the monolayer of macrophage culture. After 48 h of infection, lactate dehydrogenase (LDH) release was assessed using the CytoTox-One homogeneous membrane integrity assay kit (Promega, Madison, WI) according to the manufacturer’s instructions.

### Urease activity.

C. neoformans cells (10^8^) were incubated in 1 ml of rapid urea broth (RUH) developed by Roberts et al. (92) and adapted by Kwon-Chung et al. (93) at 30°C. After 1 to 4 h of incubation, cells were collected by centrifugation, and 100 μl of supernatant was transferred to a 96-well plate. The absorbance of the supernatant was measured at 570 nm using an EMax Plus microplate reader (Molecular Devices, San Jose, CA). The assay was performed in triplicate for each time interval.

### Melanin quantification.

C. neoformans in 10^4^, 10^5^, 10^6^, and 10^7^ cells were spotted on minimal medium agar supplemented with 1 mM l-3,4-dihydroxyphenylalanine (l-DOPA; Sigma-Aldrich, St. Louis, MO). The plates were incubated at 30°C without light. Photos were taken after 1 to 3 days of incubation on a white light illuminator. Photos of samples were always taken together with their parental strains under the same condition in order to avoid different exposure times or light adjusted by the camera. The obtained photos were then converted to greyscale using ImageJ software. The regions of the colonies were selected and the pixels of each selected region were quantified in grayscale. The relative grayscales of the colonies from samples were normalized by the grayscales of the colonies of parental strains. The representative data shown in this paper are from the cell number of 10^6^ cells and at the time point of 24 h. Three biological independent experiments were performed for each sample.

### Macrophage killing assay.

BMDM cells (5 × 10^4^ cells/well) were infected with C. neoformans (5 × 10^4^ cells) in the presence of 10 μg/ml 18B7 MAb. The culture plates were centrifuged at 1,200 rpm for 1 min to settle yeast cells on the monolayer of macrophage culture. After 24 h of infection, phagocytized cryptococcal cells were released by lysing the macrophages with sterilized water. The lysates were serially diluted, plated onto Sabouraud agar, and incubated at 30°C for 48 h for CFU determination. This experiment was performed in triplicate for each strain.

### Virulence assay in Galleria mellonella.

Galleria mellonella larvae were purchased from Vanderhorst Wholesale (Saint Mary’s, OH). Larvae were picked based on weight (175 to 225 mg) and appearance (creamy white in color). Larvae were starved overnight at room temperature. The next day, overnight cultures of C. neoformans that grew in Sabouraud broth were washed three times with PBS and diluted to 1 × 10^5^ cells/ml. Cells in 10 μl PBS were injected into the larva via the second-to-last left proleg paw with 31-gauge needles. Infected larvae were incubated at 30°C, and the number of dead larvae was scored daily until all the larvae infected with C. neoformans ancestral strains in this study were dead. Control groups of larvae were inoculated with 10 μl of sterile PBS. Experiments were repeated at least two times with experimental groups of 15 larvae at a time.

### Whole-genome sequencing and variant identification.

Genomic DNA was prepared using cetyltrimethylammonium bromide (CTAB) phenol-chloroform extraction as described previously (94). Genomic DNA was further purified using a PowerClean DNA cleanup kit (Qiagen, Hilden, Germany). Libraries were constructed using the Illumina DNA Flex library kit and were sequenced on an Illumina HiSeq 2500 instrument to generate paired 150-base reads. An average of 145× sequence depth (range, 69 to 176×) was generated for each sample.

Reads were aligned to the C. neoformans H99 assembly (95) using BWA-MEM v0.7.12 (96). Variants were identified using GATK v3.7 (97); HaplotypeCaller was invoked in genomic variant call format (GVCF) mode with ploidy = 1, and GenotypeGVCFs was used to predict variants in each strain. The workflow used to execute these steps on Terra (terra.bio) is available on GitHub (https://github.com/broadinstitute/fungal-wdl/blob/master/gatk3/workflows/fungal_variant_calling_gatk3.wdl). Sites were filtered with variantFiltration using variant annotations QD of <2.0, an FS of >60.0, and an MQ of <40.0 (QD = QualByDepth, FS = FisherStrand, and MQ = RMSMappingQuality). Genotypes were filtered if the minimum genotype quality was <50, percent alternate alleles was <0.8, or depth was <10 (https://github.com/broadinstitute/broad-fungalgroup/blob/master/scripts/SNPs/filterGatkGenotypes.py). Genomic variants were annotated and the functional effect was predicted using SnpEff v4.1g (98).

### Cryptococcal cell karyotyping.

Cell karyotypes were analyzed by quantitative PCR. qPCR primers used in this study have been published in Gerstein et al. (59). qPCRs were performed in a StepOnePlus real-time PCR system (Applied BioSciences, Beverly Hills, CA) using 20-μl reaction volumes. All reactions were set up in technical triplicate. Each reaction mixture contained PowerUp SYBR green mastermix (Applied BioSciences), 300 nM each primer, 10 ng genomic DNA from CTAB extraction, and distilled water (dH_2_O). Cycling conditions were 95°C for 5 min, followed by 40 cycles of 95°C for 15 s and 55°C for 1 min. Melt curve analysis was performed in 0.5°C increments from 55 to 95°C for 5 s for each step to verify that no primer dimers or products from misannealed primers had been amplified. Threshold cycle (*C_T_*) values were obtained using StepOnePlus software v2.3 (Applied BioSciences), where the threshold was adjusted to be within the geometric (exponential) phase of the amplification curve. Chromosome copy numbers were determined using a modified version of the classical *C_T_* method as described by Pavelka et al. (76).

### Visualization of nuclear division in pseudohyphae.

Histone 2B-GFP-tagged H99 (C1746) was allowed to interact with A. castellanii on Sabouraud agar as described above until surviving colonies with pseudohyphae emerged. The colonies were transferred to the wells of 18B7 antibody (Ab)-coated-coverslip-bottomed petri dishes with 14-mm microwells (MatTek Brand Corporation, Ashland, MA) in minimal medium. After 30 min of incubation to allow for settling down the cells, 2 ml of minimal medium was added. Images were taken every 10 min for 24 h using of a Zeiss Axiovert 200M inverted microscope with a 10× phase objective and GFP channel in an enclosed chamber under 30°C conditions.

### Stress sensitivity test.

The overnight cultures were diluted in Sabouraud broth to an OD_600_ of 2 and further diluted 10-, 10^2^-, 10^3^-, 10^4^-, and 10^5^-fold. The dilutions (5 μl) were spotted onto Sabouraud agar plates supplemented with 16 μg/ml fluconazole and incubated for 48 h at 30°C. Plates without fluconazole were also incubated for 48 h at either 30°C or 37°C.

### Growth curve.

C. neoformans strains Ftc555-1, F5, and F6 were grown in Sabouraud medium at 30°C with orbital shaker (120 rpm) for 7 days, with data measurements each 24 h. The assay was performed in a 96-well plate, and some serial dilutions were done, with a cell concentration range between 1.0 × 10^7^ to 5.0 × 10^3^/well. Each condition was carried out in triplicate. The growth was measured by optical density at 600 nm.

### Murine infection.

Six-week-old female A/J mice (JAX stock no. 000646; Jackson Laboratory) were infected intranasally with 1.0 × 10^7^ yeast cells of each C. neoformans strain used in this study. Three groups of mice (*n* = 8 animals per group) were infected, and animals were observed daily for 60 days and euthanized at any time if showing more than 20% weight loss, appearance of moribundity, pain, or inability to feed. Surviving animals after 60 days were euthanized and tissues extracted for fungal burden and cytokine level determination.

A second experimental infection was performed with some modifications. Six-week-old female A/J mice were infected intranasally with 1.0 × 10^7^ yeast cells of each C. neoformans strain (*n* = 5 animal per group) and then euthanized after 5 days. The organs were also removed for fungal burden and cytokine level evaluation.

In all of the mouse experiments, animals were intranasally infected with C. neoformans yeasts, in a total volume of 20 μl (10 μl in each nasal cavity of the mouse). Mice were anesthetized with 60 μl xylazine-ketamine solution intraperitoneally (95 mg of ketamine and 5 mg of xylazine per kg of animal body weight) to perform intranasal infection.

### Fungal burden assessment.

The fungal burden was measured by counting CFU. After animals were euthanized, the lungs were removed, weighed, and homogenized in 1 ml of PBS. After serial dilutions, homogenates were inoculated onto Sabouraud agar plates with 10 U/ml of streptomycin-penicillin. The plates were incubated at room temperature, and the colonies were counted after 48 to 72 h.

### Determination of cytokine levels in the organs.

The spleen and lungs of each mouse were macerated with protease inhibitor (complete, EDTA-free; Roche Life Science, IN, United States) and centrifuged; supernatants of these samples were used for cytokine detection by a sandwich enzyme-limited immunosorbent assay (ELISA) using commercial kits (BD OptEIA; BD Franklin Lakes, NJ, US) for the following cytokines: IL-2 (catalog no. 555148), IL-4 (catalog no. 555232), IL-10 (catalog no. 555252), IFN-γ (catalog no. 551866), and TNF-α (catalog no. 555268).The protocol was followed according to the manufacturer’s recommendations. The reading was performed in a plate spectrophotometer at 450 nm and 570 nm.

### Data availability.

All sequences for this project are available in the NCBI database under BioProject accession number PRJNA640358.

10.1128/mBio.00567-21.10 MOVIE S3 Time-lapse imaging showing nuclear division of pseudohyphae. Download Movie S3, AVI file, 0.3 MB. Copyright © 2021 Fu et al. 2021 Fu et al. https://creativecommons.org/licenses/by/4.0/ This content is distributed under the terms of the Creative Commons Attribution 4.0 International license.
