# Supplementary material for: Amoeba Predation of Cryptococcus neoformans Results in Pleiotropic Changes to Traits Associated with Virulence
Source: mBio. 2021 Apr 27;12(2):e00567-21. doi: 10.1128/mBio.00567-21 (PMC8092252; doi:10.1128/mBio.00567-21)
Supplement: TABLE S1 [file mBio.00567-21-st001.docx]

S1 Table. The list of amoeba-passaged isolates and their parental strains analyzed in this study

| Parental strain | Isolates | Description |
| --- | --- | --- |
| H99  (referred to as HP) | H1 | Amoeba-passaged isolate; Isolated from survival hyphal colony 1 |
|  | H2 | Amoeba-passaged isolate; Isolated from survival hyphal colony 1 |
|  | H13 | Amoeba-passaged isolate; Isolated from survival hyphal colony 2 |
|  | H14 | Amoeba-passaged isolate; Isolated from survival hyphal colony 1 |
|  | H16 | Amoeba-passaged isolate; Isolated from survival hyphal colony 2 |
|  | H17 | Amoeba-passaged isolate; Isolated from survival hyphal colony 2 |
|  | HC1 | Control; Isolated from a colony on the same plate with isolates but without interacting with amoeba |
|  | HC2 | Control; Isolated from a colony on the same plate with isolates but without interacting with amoeba |
|  | HC3 | Control; Isolated from a colony on the same plate with isolates but without interacting with amoeba |
| A1-35-8  (referred to as AP) | A1 | Amoeba-passaged isolate; Isolated from survival hyphal colony 1 |
|  | A2 | Amoeba-passaged isolate; Isolated from survival hyphal colony 2 |
|  | A3 | Amoeba-passaged isolate; Isolated from survival hyphal colony 2 |
|  | A4 | Amoeba-passaged isolate; Isolated from survival hyphal colony 3 |
|  | A5 | Amoeba-passaged isolate; Isolated from survival hyphal colony 3 |
|  | A6 | Amoeba-passaged isolate; Isolated from survival hyphal colony 3 |
|  | AC1 | Control; Isolated from a colony on the same plate with isolates but without interacting with amoeba |
|  | AC2 | Control; Isolated from a colony on the same plate with isolates but without interacting with amoeba |
|  | AC3 | Control; Isolated from a colony on the same plate with isolates but without interacting with amoeba |
| Ftc555-1  (referred to as FP) | F1 | Amoeba-passaged isolate; Isolated from survival hyphal colony 1 |
|  | F2 | Amoeba-passaged isolate; Isolated from survival hyphal colony 2 |
|  | F3 | Amoeba-passaged isolate; Isolated from survival hyphal colony 3 |
|  | F4 | Amoeba-passaged isolate; Isolated from survival hyphal colony 3 |
|  | F5 | Amoeba-passaged isolate; Isolated from survival hyphal colony 4 |
|  | F6 | Amoeba-passaged isolate; Isolated from survival hyphal colony 4 |
|  | FC1 | Control; Isolated from a colony on the same plate with isolates but without interacting with amoeba |
|  | FC2 | Control; Isolated from a colony on the same plate with isolates but without interacting with amoeba |
|  | FC3 | Control; Isolated from a colony on the same plate with isolates but without interacting with amoeba |
