# Supplementary figures and images for: Amoeba Predation of Cryptococcus neoformans Results in Pleiotropic Changes to Traits Associated with Virulence
Source: mBio. 2021 Apr 27;12(2):e00567-21. doi: 10.1128/mBio.00567-21 (PMC8092252; doi:10.1128/mBio.00567-21)

## Supplementary Figure 1

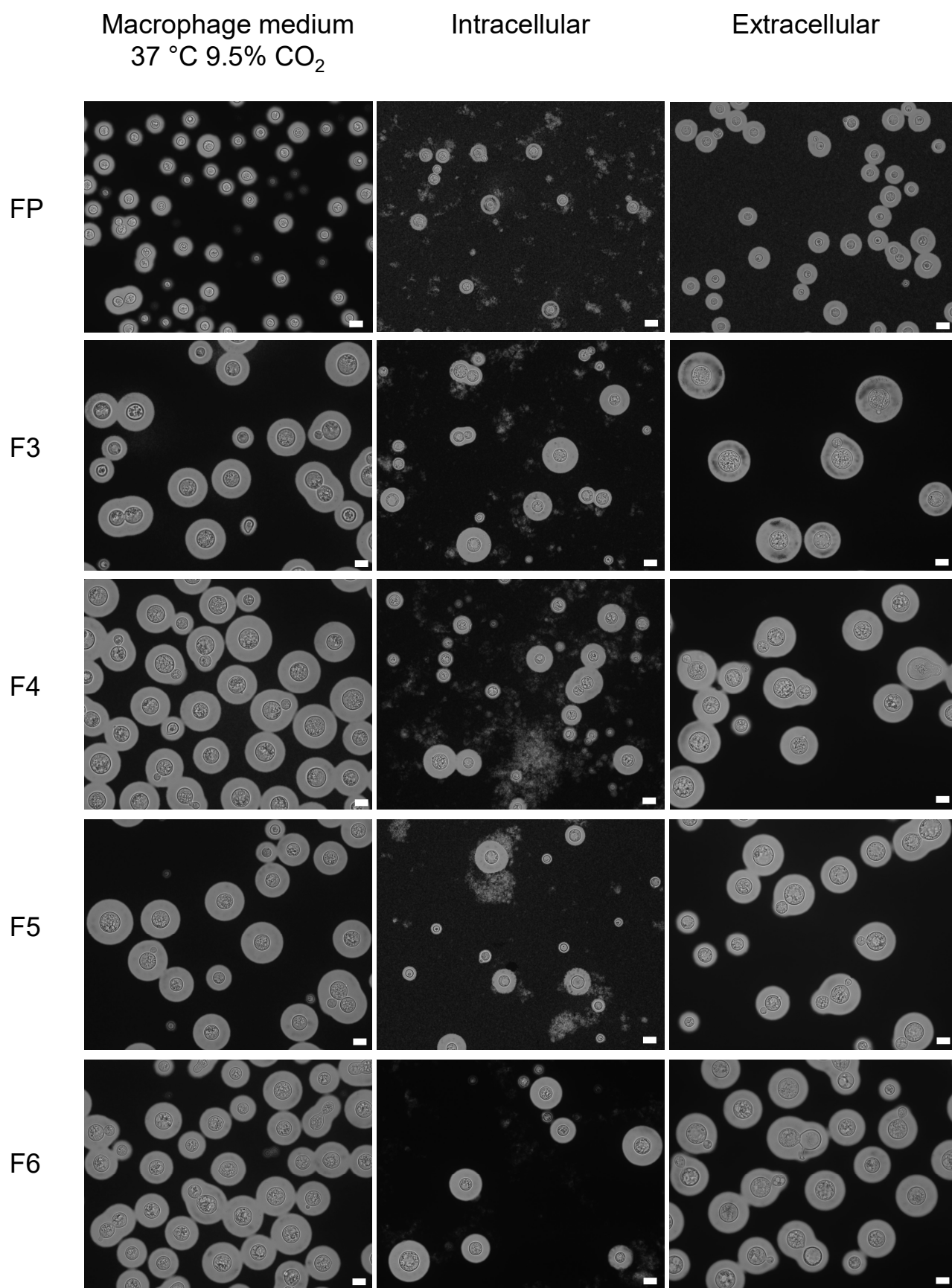

Supplement: FIG S1 [file mBio.00567-21-sf001.pdf]

# Supplementary Figure 3

**A**

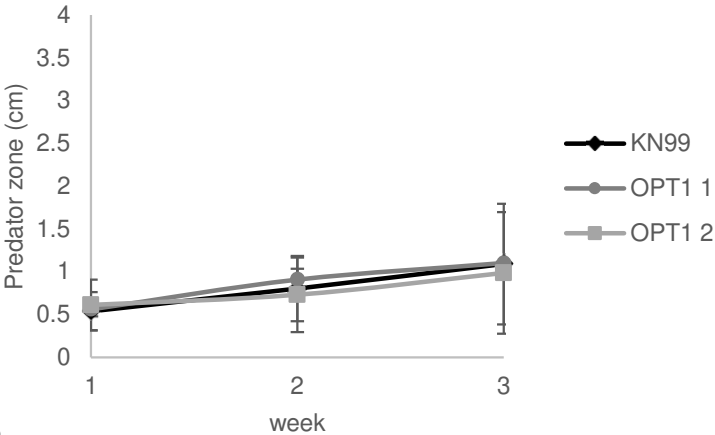

**B**

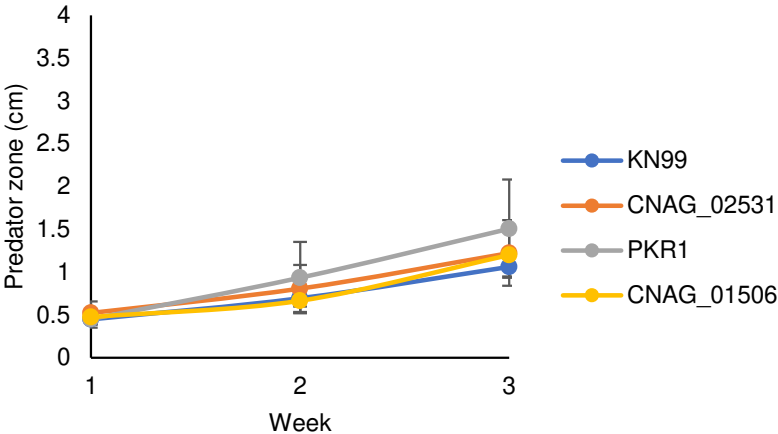

Supplement: FIG S3 [file mBio.00567-21-sf003.pdf]

Supplementary Figure 4

60 dpi

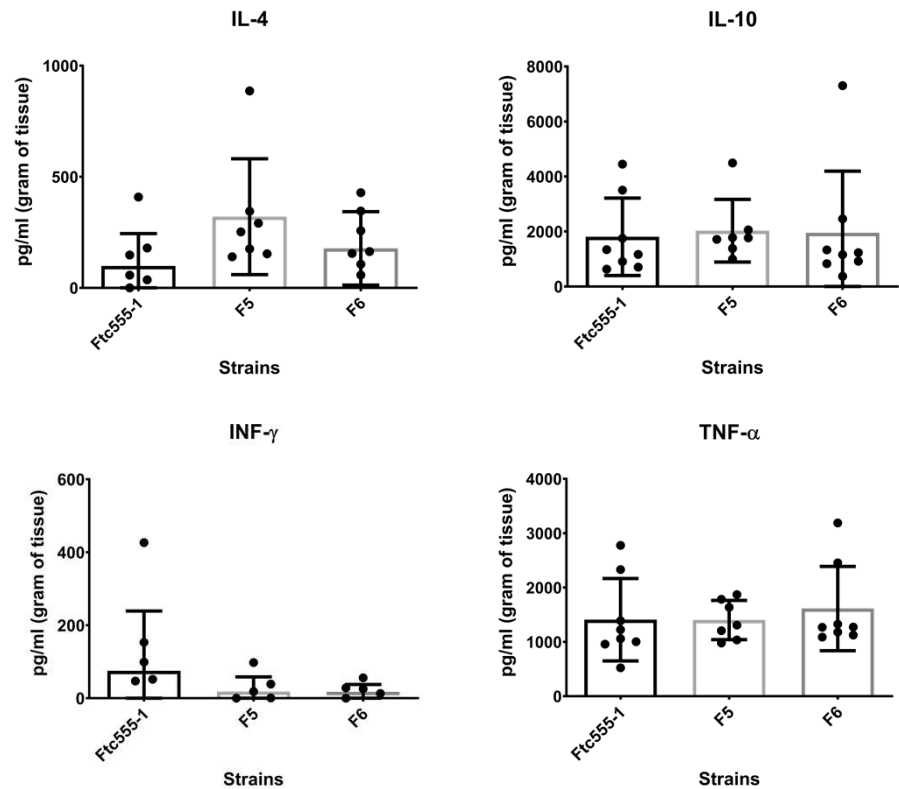

5 dpi

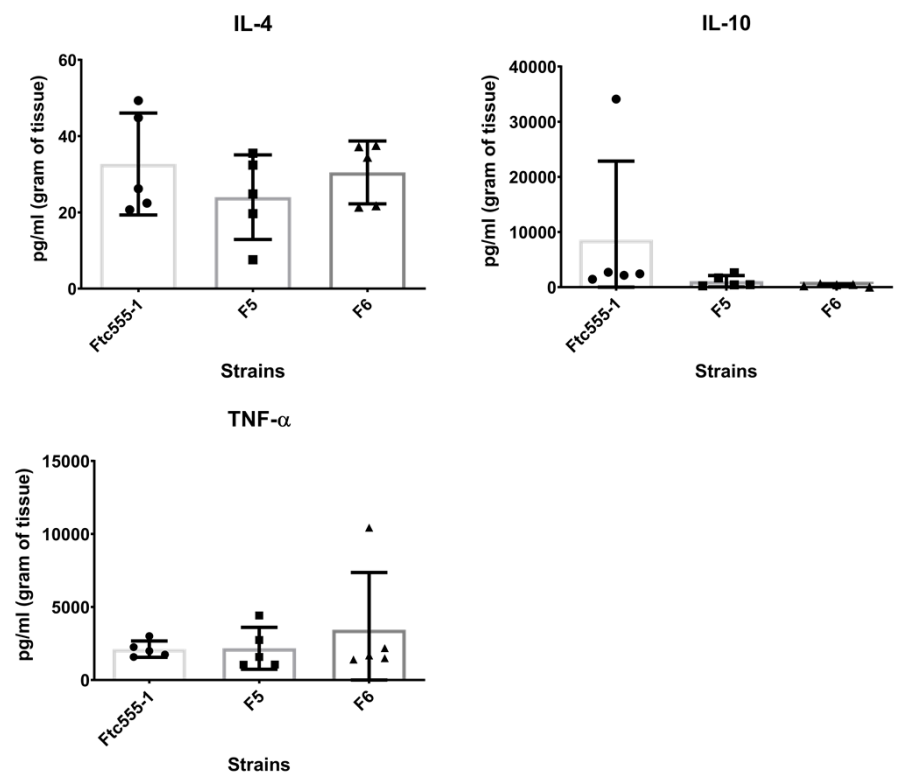

Supplement: FIG S4 [file mBio.00567-21-sf004.pdf]

# Supplementary Figure 5

**A**

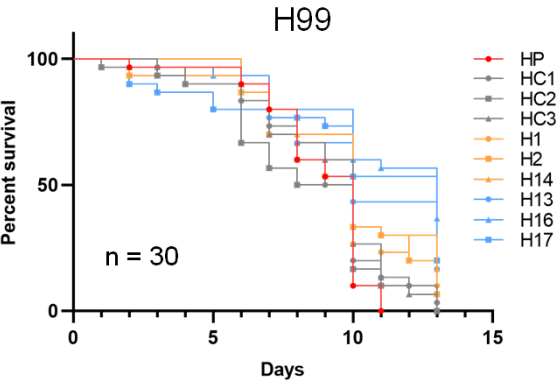

**B**

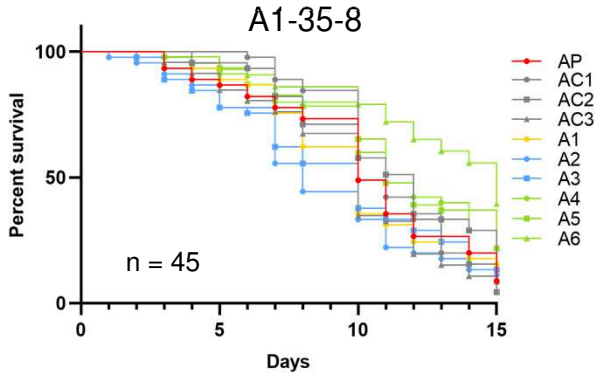

**C**

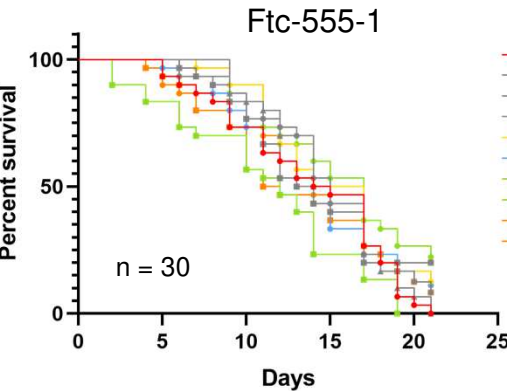

Supplement: FIG S5 [file mBio.00567-21-sf005.pdf]
