# Supplementary material for: Amoeba Predation of Cryptococcus neoformans Results in Pleiotropic Changes to Traits Associated with Virulence
Source: mBio. 2021 Apr 27;12(2):e00567-21. doi: 10.1128/mBio.00567-21 (PMC8092252; doi:10.1128/mBio.00567-21)

Supplementary Figure 2

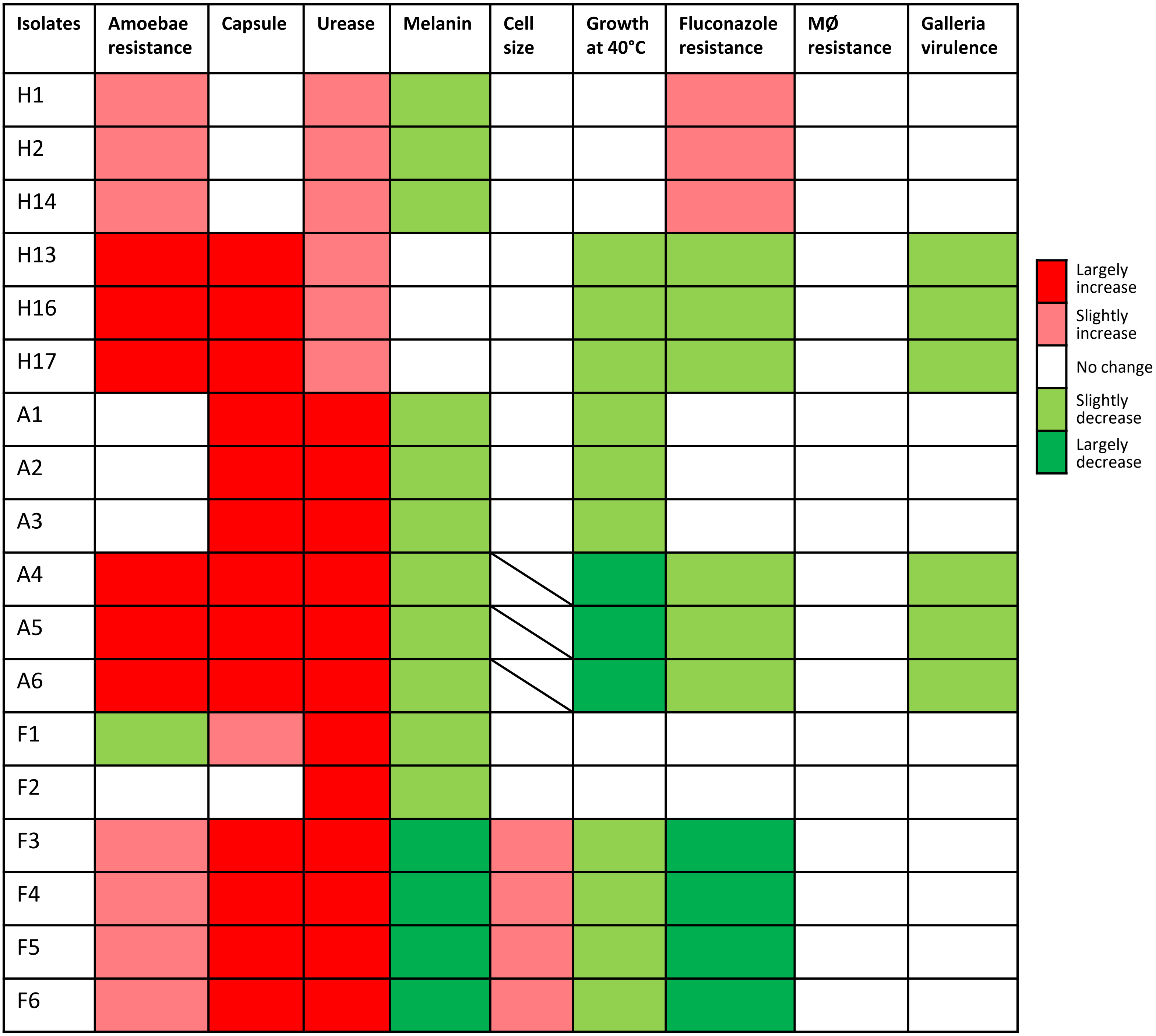

Supplement: FIG S2 [file mBio.00567-21-sf002.pdf]
