# Supplementary material for: Amoeba Predation of Cryptococcus neoformans Results in Pleiotropic Changes to Traits Associated with Virulence
Source: mBio. 2021 Apr 27;12(2):e00567-21. doi: 10.1128/mBio.00567-21 (PMC8092252; doi:10.1128/mBio.00567-21)
Supplement: TABLE S2 [file mBio.00567-21-st002.docx]

S2 table. High impact indels found in passaged Ftc555-1 isolates

| Variants | Chr | Position | Reference | Alternate | Gene ID | Gene function | Effect of mutation |
| --- | --- | --- | --- | --- | --- | --- | --- |
| F3, F4 | 1 | 2273122 | T | TC | CNAG_07391 | hypothetical protein | Frameshift at K68 |
| F3, F4 | 1 | 2273262 | G | GA | CNAG_07391 | hypothetical protein | Frameshift at P21 |
| F2, F9 | 1 | 2274716 | G | GCCAGTCGTCATCTGTGGGTAAGCTGGTGGAAATTCCAACAATAATATATATGTTAAATGAATGATTGAGGACTGGTACGGA,GCCAGTCGTCATCTGTGGGTAAGCTGGTGGAAATTCCAACAATAATATATATGTTAAATGAATGATTGAGGACTGGTACGGATGCTCAGC,GCCAGTCGTCATCTGTGGGTAAGCTGGTGGAAATTCCAACAATAATATATATGTTAAATGAATGA,GCCAGTCGTCATCTGTGGGTAAGCTGGTGGAAATTCCAACAATAATATATATGTTAAATGAATGATTGAGGACTGGTACGGATGC | CNAG_07392 | hypothetical protein | Frameshift at V38; Duplication of G11 to G37; Duplication of F10 to G37 |
| F3, F9 | 4 | 11614 | G | GATAC | CNAG_04932 | hypothetical protein | Frameshift at G478 |
| F5, F8 | 4 | 409532 | CG | C | CNAG_07810 | mitochondrial protein | Frameshift at P30 |
| F6, F8 | 4 | 748423 | TTG | T | CNAG_05212 | hypothetical protein | Frameshift at C352 |
| F1, F4, F5, F6, F7, F8, Ftc555-1 | 4 | 974409 | C | CGT | CNAG_05298 | hypothetical protein | Frameshift at K444 |
| F2 | 5 | 127671 | G | GAT | CNAG_06833 | hypothetical protein | Frameshift at G362 |
| F2 | 5 | 127672 | G | GGCAACTTCAATCAC | CNAG_06833 | hypothetical protein | Frameshift at R363 |
| F2 | 5 | 127676 | C | CGTCCCGAGCATGCTGCTGCGGATGATTTTAAGAAGGCCCCTCCCCCAATTTTCTTGCCTGCCGCAGAAGTAGATGGAGCGAGACGTGCTGTGGATAGAAAG | CNAG_06833 | hypothetical protein | Frameshift at A364 |
| F2, F3, F4, F5, F6, F7, F8, F9, Ftc555-1 | 5 | 477753 | T | TC | CNAG_01370 | hypothetical protein | Frameshift at H7 |
| F4, F9 | 5 | 1365036 | GCTCCA | G | CNAG_01041 | hypothetical protein | Frameshift at M155 |
| F3, F5 | 6 | 20941 | T | TGACGGCCTTACACGCAAGCCCACAAACTGTTTTCGCTCAGGCCCTGTTATCATCAATCAAAAGTCTCTTATCAAACATCGATACTAACGCAAGGACGGC | CNAG_02551 | dihydroxyacetone kinase 1 | Deletion of Y12 and replaced with CRPCVSIDVXXETFDXXXQGLSENSLWACVTerGRH |
| F9 | 7 | 480992 | GAAGCATC | G | CNAG_06683 | hypothetical protein | Deletion of M1 and L2 leading to start codon loss |
| F3 | 7 | 798446 | C | CAACAGTAGTGCCCAAAATCTTGAAGGCGATCTTCCTGGACATAGCCATCTCCGCGTCCCAAACTA | CNAG_05760 | endoplasmic reticulum protein | Frameshift at V93 |
| F3, F7 | 8 | 313927 | T | TTCCCCTTAGATGTGTTATCTTGCGGTACATTGTCCTTCTCGCGCAGGTTTGGTGGGGTTTTGCTGGTTTCCTCGATAATCAAAGTGTTGGGAGAGTCGATTGTGTCAGCGA | CNAG_03195 | hypothetical protein | Duplication of H207 to V243 |
| F3, F4, F5, F6, F8, Ftc555-1 | 8 | 1042000 | T | TTTTTTTTTTTTTTTTC | CNAG_07737 | hypothetical protein | Frameshift at K90 |
| F1, F2, F6, F7, F8, Ftc555-1 | 9 | 469607 | GCA | G | CNAG_07764 | hypothetical protein | Frameshift at D89 |
| F1, F7, Ftc555-1 | 9 | 469621 | T | TACTAGGAGTGTTTTAGACGCGTGGCGTTGTAAAATTTATATCGGAAGACAGTTCAG | CNAG_07764 | hypothetical protein | Frameshift at G86 |
| F6 | 9 | 469623 | C | CT,CTAGGAGTGTTTTAGACGCGTGGCGTTGT | CNAG_07764 | hypothetical protein | Frameshift at V85 |
| F4, F7, F8, Ftc555-1 | 10 | 582743 | G | GTGTTGGGAGGATGGTA,GTGTTGGGAGGATGGTAGCGAAGTGGGGATTGTGTAATATAGAAAAGAGGCCATGATGTCAGATCAA | CNAG_07998 | hypothetical protein | Frameshift at G201; Deletion of G201 and replaced with VLGGWXRSGDCVIXKRGHDVRSS |
| F4, F7, F8, Ftc555-1 | 10 | 582745 | C | CGAAGTGGGGATTGTGTAATATAGAAAAGAGGCCATGATGTCAGA | CNAG_07998 | hypothetical protein | Frameshift at S202 |
| F4, F6 | 10 | 583223 | A | AATTGTAAGTGCATAGCACTATACTCATGCATTCCCTATGCATGTATTTCATATGCATGT,AATTGTAAGTGCATAGCACTATACTCATGCATTCCCTATGCATGTATTTCAT | CNAG_07998 | hypothetical protein | Frameshift at G362; Insertion of CKCIALYSCIPYA between N361 and G362 |
| F8 | 10 | 583224 | A | ATTGTAAGTGCATAGCACTATACTCATGCATTCCCTATGCATG,ATTGTAAGTGCATAGCACTATACTCATGCATTCCCTATGCATGTATTTCATATGCATGTATT | CNAG_07998 | hypothetical protein | Frameshift at G362; Insertion of CKCIALYSCIPYAC between N361 and G362 |
| F2, F3, F4, F5, F7 | 10 | 1025227 | C | CTG | CNAG_04564 | phytanoyl-CoA dioxygenase | Frameshift at K156 |
| F2, F4, F5, F7, F8, F9 | 10 | 1025266 | G | GCGTTC | CNAG_04564 | phytanoyl-CoA dioxygenase | Frameshift at P144 |
| F2 | 12 | 582043 | C | CAGGAAGGTGGGATGAGATGAGGCATAAGGCGAGGAGGAGAGACGATGATGATG | CNAG_06193 | CMGC/RCK protein kinase | Frameshift at S879 |
| F2 | 12 | 582046 | C | CACTCAATGTCTAGCTT | CNAG_06193 | CMGC/RCK protein kinase | Frameshift at V880 |
| F1, F2, F9, Ftc555-1 | 14 | 15671 | T | TTGATAAATGAATGATTGAAGGACTGGTACGGATGCTCAGCTTCCTATCTAACCAAAGTCAGATGAGGAAAG | CNAG_05338 | hypothetical protein | Frameshift at E126 |
| F1, F2, F4, F5, F6, F7, F8, F9, Ftc555-1 | 14 | 283733 | GAT | G | CNAG_05426 | hypothetical protein | Frameshift at Y109 |
| F1, F2, F8 | 14 | 284069 | G | GTCAAGTCATTATTGACCCATCT | CNAG_05426 | hypothetical protein | Frameshift at R187 |
| F1, F2, F4, F5, F6, F7, F9, Ftc55-1 | 14 | 443464 | G | GA | CNAG_05487 | hypothetical protein | Frameshift at M39 |
| F2, F3, F4, F5, F6, F7, F9, Ftc555-1 | 14 | 444620 | T | TCACCA | CNAG_05487 | hypothetical protein | Frameshift at A333 |
